# Chemical Modification of Reducing End‐Groups in Cellulose Nanocrystals

**DOI:** 10.1002/anie.202002433

**Published:** 2020-09-09

**Authors:** Katja Heise, Gwendoline Delepierre, Alistair W. T. King, Mauri A. Kostiainen, Justin Zoppe, Christoph Weder, Eero Kontturi

**Affiliations:** ^1^ Department of Bioproducts and Biosystems Aalto University P.O. Box 16300 FI-00076 Aalto Espoo Finland; ^2^ Adolphe Merkle Institute Université de Fribourg Chemin des Verdiers 4 CH-1700 Fribourg Switzerland; ^3^ Materials Chemistry Division Chemistry Department University of Helsinki A.I. Virtasen aukio 1, P.O. Box 55 FI-00014 Helsinki Finland; ^4^ Omya International AG Baslerstrasse 42 CH-4665 Oftringen Switzerland

**Keywords:** analytical methods, mutarotation, nanostructures, polymerization, structure–activity relationships

## Abstract

Native plant cellulose has an intrinsic supramolecular structure. Consequently, it can be isolated as nanocellulose species, which can be utilized as building blocks for renewable nanomaterials. The structure of cellulose also permits its end‐wise modification, i.e., chemical reactions exclusively on one end of a cellulose chain or a nanocellulose particle. The premises for end‐wise modification have been known for decades. Nevertheless, different approaches for the reactions have emerged only recently, because of formidable synthetic and analytical challenges associated with the issue, including the adverse reactivity of the cellulose reducing end and the low abundance of newly introduced functionalities. This Review gives a full account of the scientific underpinnings and challenges related to end‐wise modification of cellulose nanocrystals. Furthermore, we present how the chemical modification of cellulose nanocrystal ends may be applied to directed assembly, resulting in numerous possibilities for the construction of new materials, such as responsive liquid crystal templates and composites with tailored interactions.

## Graphical abstract

Topochemical modifications at the reducing end of nanocellulose are a new trend in cellulose materials research. The chemistry is aldehyde‐specific and exploits the inherent directionality of the chains in the cellulose I crystal bearing aldehyde groups exclusively on one end. This Review discusses challenges related to end‐specific modifications and highlights the opportunities for nanocellulose materials.

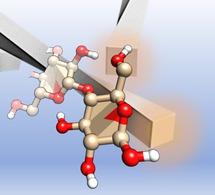

## 1 Introduction

Synthetic tailoring of natural compounds such as proteins, DNA/RNA, and lipids has unleashed a vast area of new research and technological development in materials science.^[1]^ Chemical modifications to better control particularly the nanoscale assembly of native units has proved a viable approach in many instances.^[2]^ For naturally derived carbohydrate polymers, like starch and cellulose, synthetic tools and aims have traditionally functioned within a somewhat cruder framework. A broad range of industrial starch and cellulose derivatives are being applied for thermoplastics, viscosity modifiers, and binders, to name a few.[^3^, ^4^, ^5^, ^6^]

**Figure 1:**
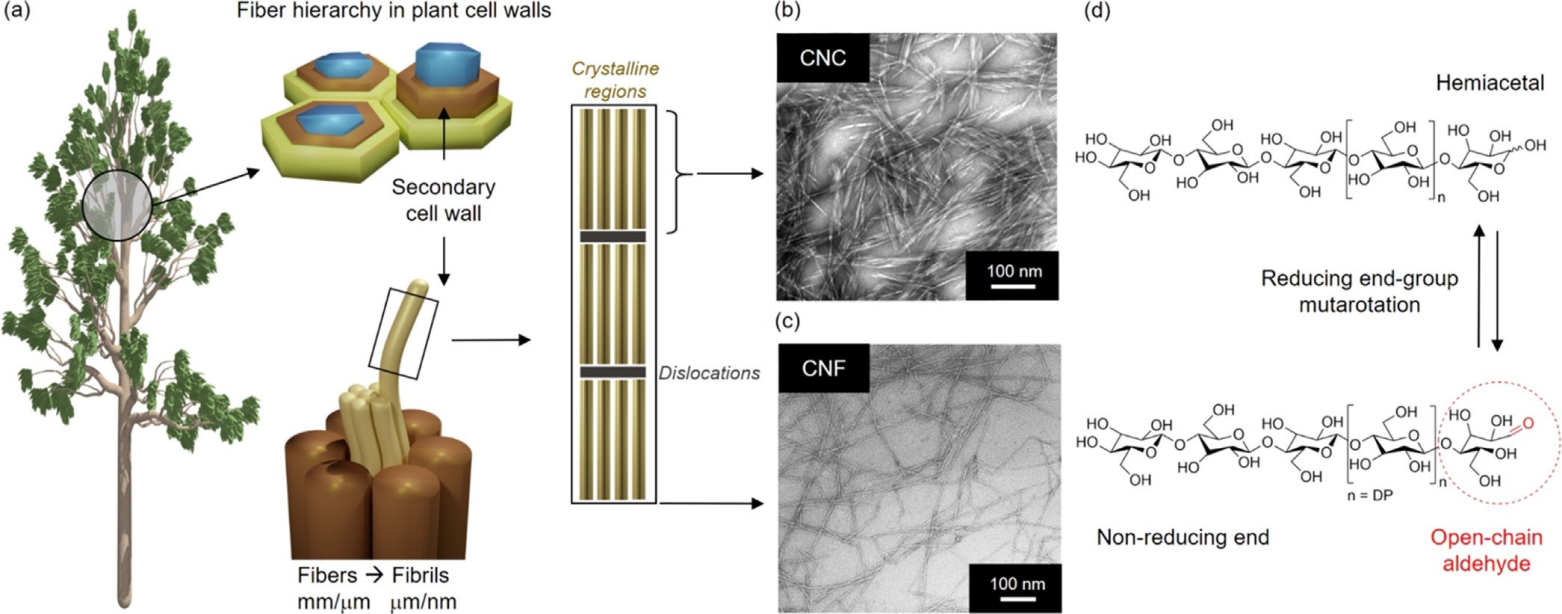
a) Schematic representation of the fiber hierarchy in plant cell walls, and the isolation of rod‐like CNCs and CNFs. b) TEM image of wood CNCs.^[12]^ Adapted with permission from ACS (2013). c) TEM image of CNF,^[13]^ modified with permission from ACS (2007). d) Molecular structure of cellulose, its reducing and non‐reducing end groups, and the tautomeric equilibrium between open‐chain aldehyde and hemiacetal.

By contrast to the mass scale production, the present century has seen the rise of nanosized objects derived from naturally occurring polysaccharides.[^7^, ^8^] The development has been markedly strong in the case of cellulose, which has been touted as a source for a new family of nanomaterials, i.e., nanocellulose.[^9^, ^10^] Indeed, the top‐down isolation of cellulose nanofibers (CNFs)^[11]^ and nanocrystals (CNCs)^[14]^ from plant‐based fibers has advanced enormously during the past decade with several semi‐industrial production sites emerging worldwide (see Supporting Information (SI), Table S1). Similarly, applications of nanocellulose have been explored both in industrial^[15]^ and academic^[10]^ realms. Although the assembly of CNFs and CNCs has been researched and utilized in sophisticated materials construction,^[10]^ routes to directed assembly by a means of specific modification have been limited. One possibility to control nanocellulose self‐assembly is the topochemically unspecific decoration of their surfaces with small molecules or macromolecules that influence the interactions among them.[^16^, ^17^, ^18^, ^19^] Recently, a trend that utilizes the directionality of the cellulose chain has surfaced in nanocellulose research. End‐wise modification exploits the reducing end unit of cellulose and the parallel alignment^[20]^ of the chains in the native cellulose I crystal, which dictates that all reducing end‐groups (REGs) are at the same terminal end of a CNC or a CNF (Figure 1).

Because of mutarotation—a phenomenon discovered by Lowry^[21]^ at the beginning of the 20th century—the familiar hemiacetal ring form of glucose is in equilibrium with an open‐chain aldehyde form (Figure 1 d). End‐wise modification simply utilizes the reactive aldehyde to introduce functional moieties at the end of cellulose chains. Particularly with nanocellulose, such end‐wise modification enables curious assemblages, relying on the asymmetric nature of the particles.^[22]^ The end‐modified CNCs can be considered as Janus‐type nanoparticles analogous to surfactants or block copolymers.

There are a number of reviews on the isolation, modification, and applications of nanocellulosic materials.[^7^, ^10^, ^16^, ^17^, ^18^, ^23^, ^24^] Yet only one review to date specifically discusses their end‐wise modification.^[23]^ In this Review we thoroughly address how the biological origin of cellulosic materials provides formidable synthetic and analytical challenges for end‐wise modification. These challenges are related to the adverse reactivity of the cellulose reducing end and the low abundance of functionalities introduced in REG‐specific reactions. The overarching purpose of this Review is to critically discuss REG modifications in the broader context of cellulose research, shedding light on synthetic aspects, mutarotation principles, and analytical horizons. Ultimately, we will speculate on how end‐modified nanocellulose structures have potential as building blocks in entirely new types of materials.

## 2 Current Aspects of Nanocellulose Materials Research

The term “nanocellulose” generally refers to anisotropic 1D objects of cellulose, isolated top‐down from plant fibers, where most components other than cellulose are removed. Additionally, nanocelluloses are synthesized bottom‐up by certain bacteria. These find applications particularly in the biomedical realm because of their high purity.^[25]^

Plant fibers consist of cellulose microfibrils: long semicrystalline threads with nanoscale diameters and lengths running up to several micrometers (Figure 1 a).[^26^, ^27^] The isolation of these microfibrils gives rise to CNFs (Figure 1 a,c). This is achieved by disintegrating the fiber by mechanical force with suitable pretreatments, such as TEMPO‐mediated oxidation or exposure to cellulose‐specific enzymes.[^28^, ^29^] On the other hand, if a strong acid is used to hydrolyze the noncrystalline regions of cellulose microfibrils, the fiber breaks down to CNCs (Figure 1 a,b).[^14^, ^24^] These rigid rods of crystalline cellulose have typical dimensions of 50–350 nm × 5–20 nm (length × width).^[30]^

In terms of fundamental research in nearly every sector, CNCs have been a more popular subject than CNFs. Because of their length and flexibility, CNFs gel at relatively low (often 0.5–1.5 wt %) concentrations.[^31^, ^32^] Rather than forming a gel, CNCs generally form chiral nematic liquid crystals (LCs) at elevated (ca. 5–10 wt %) concentrations.^[33]^ The propensity of CNCs to form specifically chiral nematic LCs was discovered in 1992^[34]^ and is still among the main lines of research on CNC‐based materials.[^35^, ^36^, ^37^, ^38^]

Concerning applications, CNFs have been envisaged for usage as the reinforcing components in composite materials^[39]^ and on their own in nonwoven networks called nanopapers.^[40]^ Another major application target has been on hydrogel‐based materials,^[31]^ particularly in the biomedical realm.^[41]^ CNCs, on the other hand, have been utilized in high‐end applications, including chiral templates[^36^, ^42^, ^43^] and stimuli‐responsive materials.[^44^, ^45^, ^46^, ^47^] This distinction is by no means strict but it arises from the intrinsic properties. CNCs are more well‐defined, both chemically and morphologically, forming stable, fluid aqueous dispersions, whereas CNFs are more heterogeneous and often also contain other plant‐based polysaccharides. As a result, the physico‐chemical properties of CNCs are often more easily interpreted than those of CNFs. Because of the low gelling point and chemical heterogeneity, also the modification of CNCs is a more established field than that of CNFs.^[17]^ For example, CNCs have been popular as substrates for graft polymerizations that transform the nanoparticles into hairy rod structures.^[19]^ It is noteworthy that not the majority, but in fact all published end‐wise modification approaches toward nanocelluloses have been targeted on CNCs. While similar modifications should a priori also be possible with CNFs, they have so far, at least to our knowledge, not been reported. Thus, CNCs are the substrates of choice for sophisticated modification techniques and the de facto nanoscale building blocks of cellulose in the current research environment.

## 3 Historical Context of Cellulose Structural Analysis

**Figure 2:**
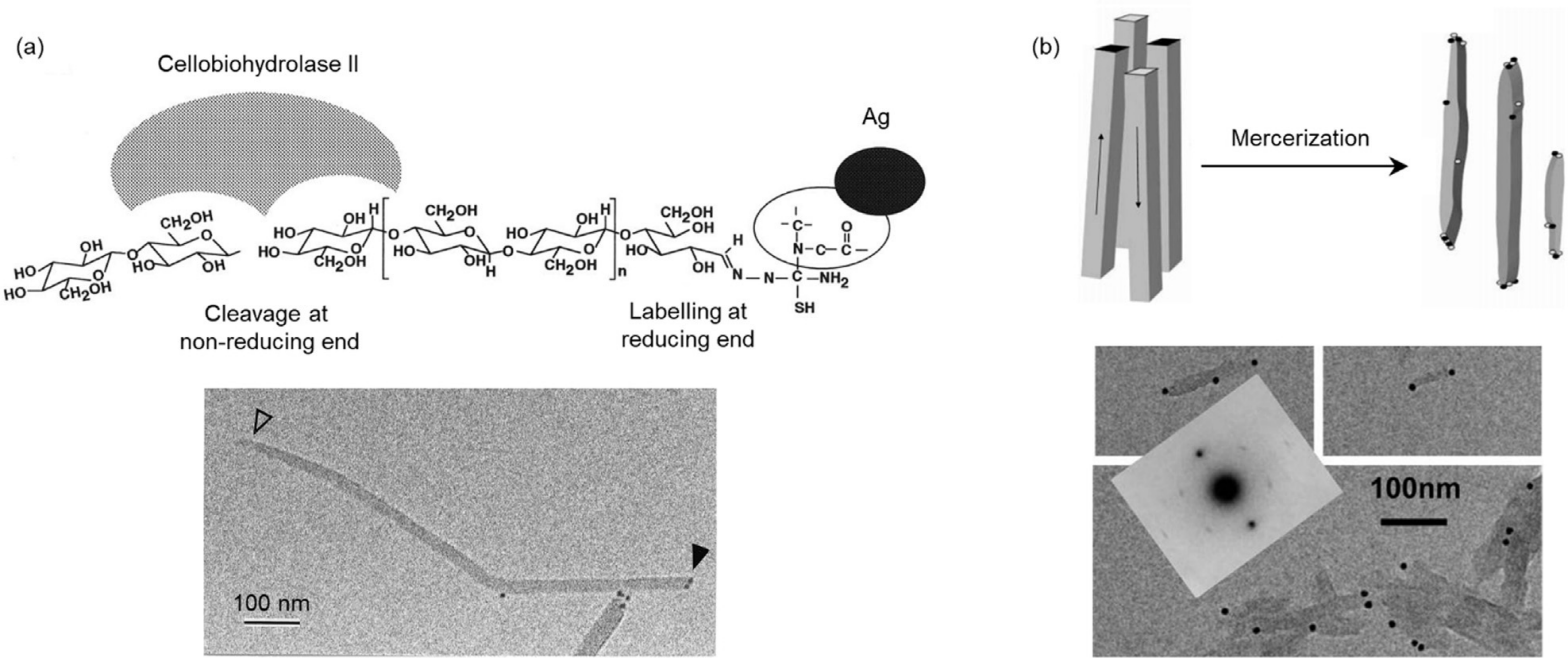
a) Top: REG labeling with Ag^0^NPs and the NREG‐specific action of cellobiohydrolase II. Bottom: TEM image of *Cladophora* cellulose with the visualized the Ag^0^‐labeled REGs (filled arrow) and the NREGs (empty arrow).^[20]^ Adapted with permission from the National Academy of Sciences (1997). b) Top: Representation of the conversion of ramie cellulose I microcrystals to cellulose II (antiparallel chains; dark dots: chain ends “up”, white dots: chain ends “down”) during mercerization. Bottom: TEM images of mercerized ramie with Au^0^NPs on both microcrystal ends.^[54]^ The molecular orientation of cellulose II was identified by electron diffraction (inset). Modified with permission from ACS (2006).

Before entering materials‐related research, end‐wise modifications were used for cellulose structural analysis, enabling a better understanding of the supramolecular structure of its different polymorphs, cellulose biosynthesis, and enzymatic degradation. The parallel orientation of the native cellulose I crystal was established in X‐ray diffraction studies throughout the 20th century.[^48^, ^49^, ^50^] However, in their electron microscopic studies in 1984, Hieta and co‐workers were the first to visualize the parallel orientation of native cellulose I chains in an algal species (*Valonia*) by labelling the REGs with silver nanoparticles (Ag^0^NPs) after their selective oxidation with NaClO_2_.^[51]^ Kuga and Brown (1988) optimized this labelling approach for Ramie and bacterial cellulose using the aldehyde‐specific ligation of thiosemicarbazide, which allowed for the topochemical attachment of a silver protein complex providing nucleation sites for Ag^0^NPs.^[52]^ This approach efficiently elucidated the inherent parallel‐up packing in both cellulose I_α_ and I_β_, supporting earlier microscopic studies. A combination of the silver staining technique with microdiffraction‐tilting electron crystallographic analysis and a selective enzymatic degradation starting from the nonreducing end‐groups (NREGs) has further provided evidence for the action of cellulose synthase complexes during cellulose biosynthesis (Figure 2 a).^[20]^ An important example of polysaccharide REG labelling for electron microscopy was demonstrated by Sugiyama and co‐workers.[^53^, ^54^] They employed biotin–streptavidin binding to attach gold nanoparticles (Au^0^NPs) on the REGs. In this way the authors could visualize the antiparallel nature of the cellulose II polymorph (Figure 2 b)—a significant entry in the literature because the antiparallel orientation of cellulose II has ignited fervor within the cellulose community for decades.

In addition to metal nanoparticles, fluorescent anthranilic acid—attached via reductive amination—has been used as a cellulose REG label to investigate the action of reducing‐end‐specific cellobiohydrolases.[^55^, ^56^]

Most of the applied synthetic strategies used to target cellulose REGs are based on well‐established chemistries with respect to the opportunities an aldehyde group provides.

The efficiency and selectivity of the mechanisms behind REG modifications have been widely demonstrated in carbohydrate analysis and bioconjugation. However, the steric constraints and the extent of mutarotation set definite limitations for the modifications of cellulose REGs. Therefore, before discussing the materials perspective of cellulose end‐wise modification, we will elaborate on the principal mechanisms and reaction conditions used to target the REGs, followed by a treatise on mutarotation.

## 4 Mechanistic Notes

**Figure 3:**
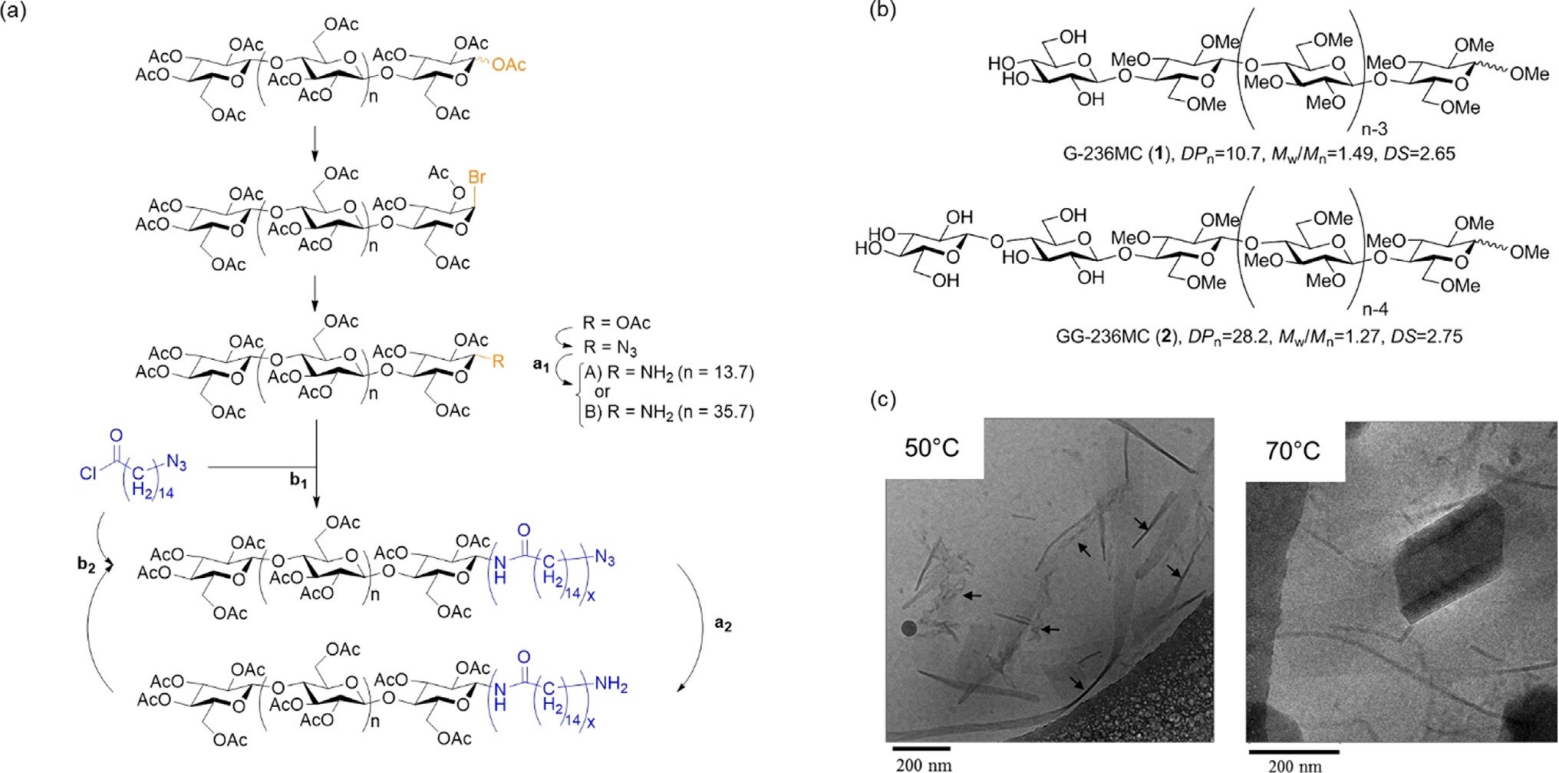
Diblock copolymers based on end‐modified cellulose derivatives: a) Pathway for the synthesis of cellulose triacetate‐*block*‐oligoamide‐15, starting from the REG of cellulose triacetate.^[57]^ b) Chemical structures of diblock copolymers consisting of hydrophilic glucosyl or cellobiosyl and hydrophobic 2,3,6‐tri‐*O*‐methyl‐glucopyranosyl blocks.^[58]^ c) Cryo‐TEM images of self‐assembled nanostructures formed from G‐236MC (**1**) at 50 °C (ribbon‐like) and 70 °C (ribbon‐like and multilamellar).^[58]^ Reproduced with permission, Copyright ACS (2012).

Most synthetic approaches toward modifying the REGs of cellulosic substrates rely on aldehyde‐specific chemistry. A very different tactic was established by the groups of Kamitakahara and Enomoto‐Rogers: the synthesis of diblock copolymers starting from cellulose triacetate (CTA) with an REG‐selective deacetylation under homogeneous (solution‐state) conditions in chloroform.[^57^, ^59^, ^60^, ^61^] Via a multistep grafting‐to pathway (Figure 3 a), they succeeded in synthesizing cellulose‐based copolymers, bearing hydrophobic tails, that self‐assembled into amphiphilic nanoparticles. Kamitakahara and co‐workers also demonstrated the synthesis of amphiphilic diblock copolymers based on regioselectively methylated celluloses.[^58^, ^62^] The combination of sulfuric acid catalyzed methanolysis and glycosylation via the trichloroacetimidate method was thereby the key toward well‐defined diblock copolymers (Figure 3 b).^[62]^ Depending on their structure (degree of polymerization (DP), substitution pattern), these copolymers showed a temperature‐triggered self‐assembly into ribbon‐like and multilamellar nanostructures (Figure 3 c).^[58]^ A profound structural confirmation of the end‐to‐end conjugated building blocks was provided by solution‐state ^1^H and ^13^C NMR spectroscopy, combined with MALDI‐TOF MS for molar mass determinations.^[62]^

CNC end‐wise modifications are exclusively carried out as heterogeneous (dispersion‐state) reactions, i.e. modifications that are restricted to surface‐accessible reaction sites. The surface chemistry of CNCs plays thereby a crucial role for the reaction selectivity and the overall conversion. In most contributions on CNC end‐wise modification, the CNCs were isolated by H_2_SO_4_ hydrolysis (see SI, Table S2), which partially substitutes the nanocrystal surface with charged sulfate half‐ester groups (‐OSO_3_^−^).^[63]^ Yet there are no definitive analytical accounts of the preferred localization of sulfate half‐esters on the CNC surface, although NMR studies on CNCs from H_3_PO_4_ hydrolysis have proven a preferred substitution of PO_4_ esters at the C2 and/or C3 positions.^[64]^ Surface charges, in general, facilitate the colloidal stabilization of CNCs in aqueous media preventing aggregation. The surface chemistry of CNCs, besides their physical dimensions, further corresponds to the (ligno)cellulosic source. Only traces of surface impurities, e.g., residues from hemicelluloses or lignin, can adversely affect REG‐specific reactions, making a thorough surface purification like extraction imperative.^[65]^

**Figure 4:**
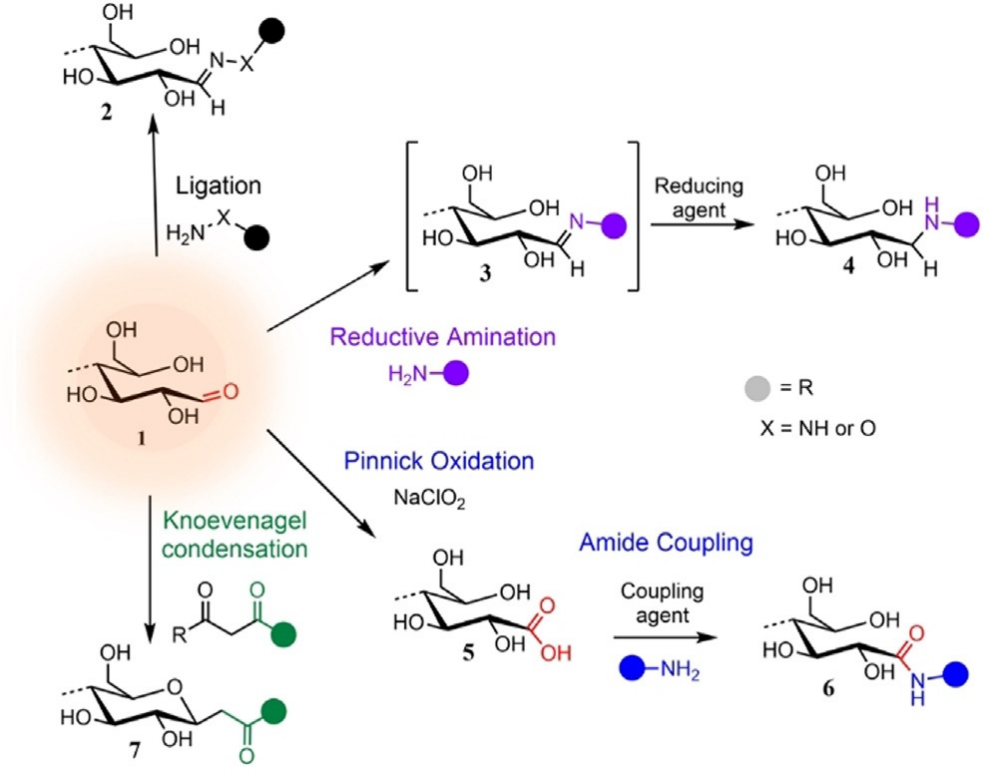
Main mechanisms used to target cellulose REGs (**1**): Ligation of hydrazine analogues (NH_2_‐NH‐R) or hydroxylamine derivatives (NH_2_‐O‐R) affording hydrazones or oximes (**2**); reductive amination forming an imine intermediary (**3**) that is reduced to a stabilized secondary amine (**4**); Pinnick oxidation affording carboxylated REGs (**5**) that allow for subsequent amidation (**6**); Knoevenagel condensation affording a *C*‐glycoside ketone (**7**).

The most common aldehyde‐specific approaches toward modifying the REGs of cellulosic substrates, including CNCs, are shown in Figure 4. In this Review, we classify them as hydrazine, hydroxylamine, and thiosemicarbazide ligation (**2**), reductive amination (**3**/**4**), Pinnick oxidation (**5**) followed by amidation (**6**), and Knoevenagel condensation (**7**). These reactions are mostly used as the initial step to activate the anomeric center for, e.g., subsequent polymer grafting or click chemistry. For a better overview, Table 1 summarizes the different homogeneous and heterogeneous reaction setups applied to (nano)cellulose REGs.

**Table 1:** Main reaction types used to modify cellulose REGs and typical reaction conditions covering homogeneous (*) and heterogeneous systems.

Reaction type	Reactants	Medium, pH conditions/catalysis	Temperature, duration	Ref.
ligation	aliphatic/aromatic hydrazine analogues^[a]^	aqueous, (mostly) alkaline	25–35 °C, 24–72 h	[76, 77, 78, 79, 80]
		DMSO (anhydrous), Ar atmosphere	50–60 °C, 4 days	[109]
				
	hydroxylamines	aqueous, acidic or DMAc/LiCl (2.5 % w/v), Et_3_N*	40 °C, up to 700 h	[73, 74, 75]
	thiosemicarbazide	aqueous, acidic	60–65 °C, 90 min	[20, 52]
		NMMO/water (80/20, v/v)*	60 °C, 90 min	[81, 82, 83]
				
reductive amination	1° amine ligands + reducing agent	aqueous buffer, acidic or alkaline	70 °C, 24–72 h	[87, 88, 89, 90]
		MeOH/acetic acid	50 °C, 12–16 h	[54]
		DMF	RT or 70 °C, 1 or 3 days	[110]
		DMAc/LiCl*	70 °C, 24 h	[111]
				
Pinnick oxidation & amide coupling	1) NaClO_2_	1) aqueous, acidic	1) RT, 20–48 h	[22, 88, 89, 103, 104, 105]
	2) 1° amine ligands + coupling agents	2) aqueous, pH switch to alkaline	2) RT, 2 h–3 d	[88, 89, 103, 104, 105]
		aqueous, pH 5	4 °C, 24 h	[22]
				
Knoevenagel condensation	1,3‐diketones	aqueous bicarbonate, pH 8.5	80–90 °C, 4–48 h	[75]

[a] Characterized by hydrazine core (NH_2_‐NH‐R).

### 4.1 Ligation

Reactions of aldehydes with hydrazines (H_2_N‐NH‐R) and hydroxylamines (H_2_N‐O‐R) (Figure 4) are fast and simple, and their structural versatility has resulted in their immense popularity in, e.g., analytical chemistry,^[66]^ polymer synthesis,^[67]^ and bioconjugation.^[68]^ Besides, the newly formed linkages provide another attractive attribute: hydrazones and oximes (**2**) hydrolyze under mildly acidic conditions (pH 5–6), with a longer half‐life for oximes.^[69]^ This “reversibility” and the fact that they are still more stable than imines makes them highly attractive, especially in the biomedical realm (e.g., controlled drug‐release[^70^, ^71^]). Stabilization of these linkages, in turn, has been achieved by a subsequent reduction.^[72]^

Reactions with hydrazine/hydroxylamine derivatives are commonly carried out at acidic or neutral pH at ambient or elevated temperatures. Depending on the reactants molecular structure, aqueous or nonaqueous conditions are suitable.[^66^, ^68^] For the modification of cellulose REGs in heterogeneous approaches, both acidic (acetate buffer, pH 4.0)[^73^, ^74^, ^75^] and alkaline (borate buffer, pH 9)[^76^, ^77^, ^78^, ^79^, ^80^] conditions have been used. A homogeneous approach in *N*,*N*‐dimethylacetamide (DMAc)/LiCl was optimized by Röhrling et al. where trimethylamine was identified as a suitable catalyst.^[73]^

Semicarbazides, including thiosemicarbazide (TSC), react with aldehydes in similar efficiency and selectivity and their applicability for cellulose REG modification has been widely demonstrated. Reactions with TSC have been carried out in aqueous acidic media (heterogeneous state)[^20^, ^52^] and in *N*‐methylmorpholine‐*N*‐oxide (NMMO) water mixtures (in solution) (Table 1).[^81^, ^82^, ^83^]

### 4.2 Reductive Amination

Reductive amination (RA) is the method of choice for the synthesis of *N*‐glycoconjugates,^[84]^ and has been used as a powerful tool toward sophisticated carbohydrate‐based structures starting, e.g., from REGs^[85]^ or other carbonyl groups^[86]^ of polysaccharides. The chemistry is simple involving two principle steps that readily proceed in water (Figure 4): imine formation (**3**) between a carbonyl and an amine (1° or 2°), and selective imine reduction to the stabilized amine (**4**).

Direct reductive aminations using hydride reducing agents, as mostly applied to cellulose REGs (Table 1),[^87^, ^88^, ^89^, ^90^] circumvent a potential instability of the imine by combining imine formation and reduction in a one‐pot reaction.^[91]^ However, this direct methodology requires the prudent choice of the reducing agent and precise control over the reaction conditions as the selectivity of imine over aldehyde reduction governs the overall reaction selectivity and yield.^[92]^ As a common reducing agent, sodium cyanoborohydride selectively reduces imines at pH 6–8,^[84]^ but the toxicity of the system has overshadowed its efficiency and controllability.^[93]^ As nontoxic alternatives, sodium triacetoxyborohydride (STAB‐H)[^94^, ^95^] and 2‐picoline‐borane (2‐Pc)[^96^, ^97^] have been successfully applied to carbohydrates, enabling selective reduction under mild aqueous and nonaqueous conditions. To identify suitable conditions for the reductive amination at CNC REGs, Arcot et al. systematically compared the use of NaBH_3_CN, STAB‐H, and 2‐Pc at different pH conditions in water.^[87]^ Accordingly, with minimal differences, all three reducing agents gave the highest conversion at pH 9.2, which corresponds to the p*K*_a_ of the amine.

### 4.3 Pinnick Oxidation and Amide Coupling

**Figure 5:**
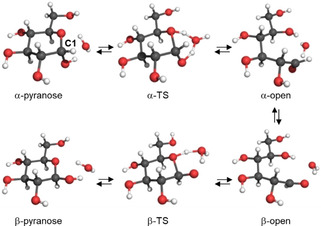
The concerted mechanism of glucose mutarotation assisted by one water molecule in the transition state (TS).^[126]^ Reproduced with permission, Copyright Elsevier Ltd (2006).

Acidic‐oxidative treatments with sodium chlorite (NaClO_2_) are well‐established for the selective delignification of cellulosic pulps[^98^, ^99^] and as a post‐treatment after, e.g., periodate oxidation converting dialdehydes to dicarboxylic acids.[^100^, ^101^] If the aldehyde oxidation is selective, the process is referred to as Pinnick oxidation (Figure 4, **5**).^[102]^ Chlorite oxidations are mild (room temperature (RT), pH 3–3.5; Table 1) and easily carried out in water.[^22^, ^88^, ^89^, ^103^, ^104^, ^105^] Risteen et al. made an attempt to determine the degree of CNC end‐group oxidation using the bicinchoninic acid (BCA) assay, which is based on the reduction of Cu^II^ to Cu^I^ by residual REG aldehyde groups (Section 7.3).^[104]^ According to their results, 57 % of the REGs were converted to carboxylic acids after a reaction time of 20 h at RT.

**Figure 6:**
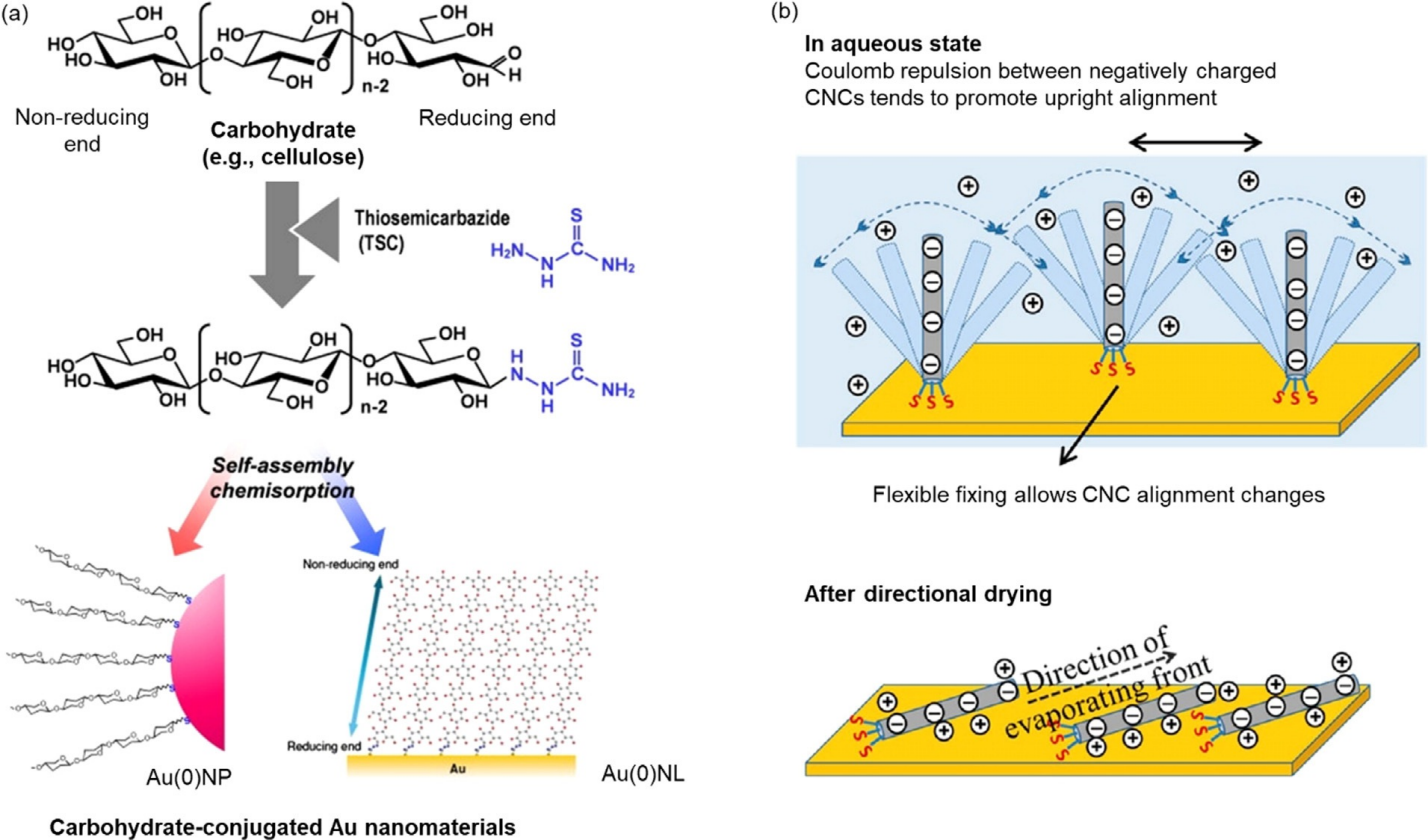
a) SAMs of TSC‐modified cellulose following their spontaneous chemisorption to gold nanoparticles (Au^0^NP) or gold nanolayers (Au^0^NL).^[136]^ Reproduced with permission, Copyright Elsevier B.V (2010). b) Representation of cilia‐mimetic surfaces based on SH‐functionalized CNCs chemisorbed to a gold substrate.^[87]^ The flexibility of S−Au bonds was demonstrated after directional drying and the realignment of the modified CNCs. Reproduced with permission, Copyright ACS (2013).

In terms of reactivity for a subsequent modification, especially in water, the REG oxidation is perhaps not the most straightforward approach and typically requires an activation of the carboxylic acids. On cellulose REGs, this activation has been realized with carbodiimide coupling agents, also referred to as “zero‐length crosslinkers” which allow for amide formation in water (Figure 4, **6**).[^22^, ^88^, ^89^, ^103^, ^104^, ^105^] Among them, EDC (1‐ethyl‐3‐(3‐dimethylaminopropyl)carbodiimide) is probably the most common one owing to its excellent water‐solubility and high reactivity toward carboxylic acids under mildly acidic (pH 4.5), aqueous conditions.[^106^, ^107^] Hydrolysis in water, however, of the active *O*‐acylisourea conjugate leads to the particular regeneration of the carboxyl group despite the high reactivity of EDC, under acidic conditions.^[108]^ To stabilize and improve the carboxyl activation in water, a second crosslinker—*N*‐hydroxysuccinimide (NHS)—has been combined with EDC forming a more stable NHS‐ester with the carboxyl group.[^106^, ^107^]

Both EDC and the EDC/NHS combination have been used to link amines to reducing‐end carboxylated CNCs.[^88^, ^89^, ^103^, ^104^, ^105^] Alternatively, a combination of dicyclohexylcarbodiimide (DCC) and pyrrolidinopyridine (4‐PPY) has been used to activate carboxylated CNC REGs.^[22]^

### 4.4 Knoevenagel Condensation

**Figure 7:**
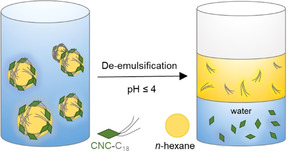
Oil‐in‐water Pickering emulsion stabilized by amphiphilic CNCs, and the pH‐triggered de‐emulsification after cleavage of the hydrazone‐conjugated alkyl chains.^[80]^ Modified with permission from ACS (2018).

Heise et al. recently demonstrated that the bicarbonate‐catalyzed condensation of 1,3‐dicarbonyl compounds with aldehydes in water can be translated to the REGs of CNCs.^[75]^ The chemistry behind this so‐called Knoevenagel condensation is well‐established.^[112]^ Applied to CNCs, it enables a mild, aqueous, one‐pot pathway toward nanocellulose *C*‐glycoconjugates (Figure 4, **7**). The reactive species attacking the REG under weakly basic conditions is an enolate anion, emerging from the deprotonation of the acidic α‐carbon of a dicarbonyl. The initial condensation is then followed by water elimination with cyclization to a *C*‐glycoside intermediate that finally undergoes retro‐Claisen aldolization forming the new *C*‐glycoside ketone.^[113]^ Applied to mono‐ and disaccharides, Knoevenagel condensations gave excellent yields and stereoselectivity (e.g., d‐cellobiose: 93 % conversion (100 % β‐anomer) with acetylacetone, in 12 h at 90 °C).[^113^, ^114^] Moreover, the mechanism can be translated to more complex compounds such as *C*‐glycolipids,^[115]^ or the newly formed glycoside ketone can be selectively targeted in a subsequent modification. The latter aspect has been suggested to be particularly beneficial in terms of controlled stereoselectivity, e.g., for subsequent aryl hydrazine ligations,^[116]^ and with respect to the fixed, closed‐ring conformation of the glycoside ketone preventing mutarotation.

The modifications of CNC REGs with acetylacetone,^[75]^ under comparable conditions to those applied to glucose or cellobiose,^[113]^ led to reducing‐end conversions of maximum 12.5 mol % after a reaction time of 48 h at 80 °C. Mutarotation and the complexity of modifying the surface of a highly crystalline substrate might be the reasons for the significantly lower conversion of CNC REGs compared to small saccharides. According to what we know from studies on monosaccharides, the reaction kinetics of REG modifications are determined by mutarotation.^[21]^ In order to understand how this tautomeric equilibrium affects reducing end‐specific reactions, its principles, illustrated by the example of well‐known studies on monosaccharides, will be discussed in Section 5.

## 5 Reducing‐End‐Group Mutarotation

The tautomeric equilibrium between hemiacetal and aldehyde generally favors the aldehyde form. However, in the case of reducing sugars, the situation changes when there are neighboring OH‐groups in the γ‐ or δ‐position (C1,C5 or C1,C4).[^117^, ^118^] The open‐chain aldehyde, thus, easily forms cyclic five‐ or six‐membered α‐ and β‐anomers by an acid–base‐catalyzed proton transfer. The hemiacetal is thereby considered as a passive tautomeric phase[^119^, ^120^] and the ring‐opening as the rate‐limiting step.^[121]^

Mutarotation has been widely explored for monosaccharides and numerous studies have demonstrated that its rate and the tautomeric composition vary with the structure of the sugar and the solution conditions (i.e. temperature, pH, and solvent type).^[122]^

The effect of temperature is obvious, with a primary impact on the mutarotation kinetics, as widely demonstrated in water^[123]^ and water–cosolvent mixtures.[^124^, ^125^] However, even at high temperatures in water, the equilibrium proportion of the free aldehyde remains very low (e.g., 0.02 % glucose aldehyde at 80 °C^[123]^). Furthermore, the temperature affects the anomeric ratio in water, following the exothermic reaction in the transition between α‐ and β‐anomer.^[125]^

Mechanistic theories and observations become more complex, when it comes to the solvent system and the catalysis. The solvent structure determines the conformational stabilization favoring, e.g., the pyranose (in water) or furanose (in dimethylsulfoxide) form of glucose.^[117]^ Moreover, the presence of water or other acid–base catalysts determines the activation mechanism of mutarotation. This can be either solvent‐catalyzed, directly involving water in a cyclic transition state, or based on a catalyst proton transfer.[^117^, ^124^, ^125^] Various hypotheses exist on how water assists mutarotation, including the mechanism proposed by Silva et al. with one water molecule acting in a concerted reaction (Figure 5).^[126]^ Without added catalyst, an increasing concentration of nonaqueous cosolvent reduces the mutarotation rate, as demonstrated by experimental data.[^124^, ^125^] The general effect of acid–base catalysis on the mutarotation has been extensively studied, with early contributions by Lowry^[21]^ and Brønsted.^[127]^ Both acids and bases effectively promote mutarotation and their catalytic activity increases with their strength. Bases have been found to be generally more efficient than their conjugate acids.^[122]^ However, studies on their catalytic rate in aqueous solutions are complicated, as the ionized glycosate ion itself is catalytically active.^[128]^ The catalytic action of phenol–pyridine mixtures in nonaqueous media has led to the assumption that organic acids and bases act simultaneously in a concerted mechanism enabling proton transfer in the absence of water.^[129]^

Besides catalytic effects, the solution pH determines the position of the anomeric equilibrium, based on the p*K*_a_ difference of α‐ and β‐anomers. This pH‐dependence has been demonstrated for glucose (β‐preference in alkaline pH)^[130]^ and for other aldoses.^[131]^

How can we now translate the knowledge on the mutarotation of monosaccharides to a complex polysaccharide like cellulose? Especially in heterogeneous reactions, hierarchical orders in crystalline cellulose regions, maintained by hydrogen bonds, likely influence the conformational stabilization and the hydration of the end‐standing, β‐d‐(1→4)‐linked AGUs in water. If a native cellulose chain would hypothetically be fully solvated, the packing of water molecules around the reducing hydroxyl groups might be comparable to their arrangement around glucose, as suggested already in contributions from 1958.[^132^, ^133^] Only the glycosidic linkage would force a distortion of the end‐standing AGU in relation to the neighboring unit, enabling its conformational stabilization in the water lattice and the formation of hydrogen bonds to water molecules.[^133^, ^134^] In crystalline cellulose, the chains are packed in planar sheets^[135]^ and all hydroxyl groups, including those of the REGs, are involved in hydrogen bonding. This aspect may have a significant effect on the mutarotation kinetics and demands for an activation and liberation of the anomeric center. Whether the involvement in hydrogen bonding affects the overall accessibility of cellulose REGs is not clear, and further studies of this aspect are needed. For example, the efficiency of a catalyst other than water on mutarotation^[129]^ should be systematically investigated.

## 6 Materials Perspective of End‐wise Modifications

The translation of aldehyde‐specific mechanisms to CNCs has opened up new avenues in nanoengineering. While examples for CNCs whose REGs were modified in a topochemically controlled manner are still limited, the available studies have led to fascinating observations and pave the way toward new functional interfaces. Moreover, the selective end‐group functionalization provides an alternative to the far more established uniform modifications, preserving the unique surface‐chemistry of the nanocrystals. Accordingly, this chapter highlights the exceptional potential of end‐modification in advanced materials applications.

### 6.1 Interfacial Engineering

Kitaoka and co‐workers pioneered the exploitation of the inherent anisotropy of the cellulose chain for the design of functional, self‐assembled monolayers (SAMs) (Figure 6 a).^[81]^ Their approach was both simple and trendsetting, using the spontaneous conjugation of a topochemically thiolated cellulose to gold substrates. Thiolation was thereby realized through a TSC ligation in an 80 % NMMO solution. The conjugation of the derivative to gold resulted in a vectorial chain immobilization in which the parallel packing of the naturally occurring cellulose I crystal was replicated because of the topochemical thiolation. In their follow‐up studies, the group demonstrated the biofunctionality of SAMs prepared from cellulose and its methyl derivative in terms of carbohydrate–cell interactions, which has been attributed, e.g., to the specific surface morphology.[^83^, ^136^] As an additional feature, the thiolated methyl cellulose endowed the gold surfaces with thermally responsive wetting characteristics, on account of the reversible gelation behavior of the modified cellulose at temperatures above its lower critical solution temperature (LCST).^[82]^ Moreover, the group established the one‐pot synthesis of glyco‐decorated Au^0^NPs starting from the novel redox system NMMO/HAuCl_4_ containing TSC‐cellulose.^[137]^

Lokanathan et al. were the first to translate the concept of cellulose–gold SAMs to CNCs, showing a directed assembly of end‐thiolated nanocrystals onto gold surfaces (Figure 6 b).^[87]^ REG thiolation was realized via a reductive amination as a new synthetic approach. A surface‐sensitive approach using a quartz crystal microbalance with dissipation (QCM‐D) provided evidence for a certain flexibility of the S−Au bond, thus demonstrating a cilia‐mimetic behavior. The synthetic route toward end‐group‐thiolated CNCs was complemented in their follow‐up work in 2014, in which they succeeded in functionalizing the REGs by a milder acid chlorite oxidation and subsequent carbodiimide‐catalyzed coupling of 6‐amino‐1‐hexanethiol.^[88]^

Besides solid interfaces, REG modifications have been used for tuning the assembly of CNCs at liquid–liquid interfaces as, e.g., in Pickering emulsions (PEs). In fact, all colloidal‐sized particles adsorb to oil–water interfaces for energy minimization.^[138]^ The term “tuning” refers to the compatibilization of the hydrophilic CNCs with the oil phase—typically realized via an unselective surface‐hydrophobization[^139^, ^140^]—which improves the stability of the CNC–interface assembly and, subsequently, allows for the use of CNCs in water‐in‐oil (w/o) PEs. In the realm of w/o PEs, Du et al. have aimed at amphiphilic CNCs via an end‐specific conjugation of stearic acid (C_18_) in a two‐step fashion: 1) activation of the REGs with hydrazine, and 2) conjugation of stearic acid to the hydrazine‐modified REGs via EDC/NHS‐coupling in THF.^[80]^ As an additional feature, the modified CNCs showed a “de‐emulsification on demand function” triggered by the cleavage of the acid‐sensitive hydrazone linkages at pH<4 liberating the alkyl chains (Figure 7).

Tang et al. used a grafting‐to approach to create amphiphilic CNC–g‐poly(styrene) nanoparticles analogous to surfactants, for enhanced oil‐in‐water (o/w) emulsion stabilization.^[110]^ Starting from amine‐terminated poly(styrene), topochemical grafting was attempted by a reductive amination in DMF. However, it is unclear how mutarotation was catalyzed, suggesting a potential adsorption of the poly(styrene) on the CNC surface rather than a covalent ligation to the REGs.

### 6.2 Janus‐Type Colloids

Janus colloids are typically classified as particles in the nanometer or micrometer range displaying at least two distinctly different physico‐chemical properties on their surfaces. This includes, e.g., amphiphilic^[141]^ or dipolar^[142]^ colloids and Janus microgels.^[143]^ Starting from a solid nanoparticle, asymmetric modifications have been realized, e.g., from Pickering emulsions, enabling a whole range of phase‐selective chemistry.[^144^, ^145^] Owing to their anisotropy, Janus‐type colloids have gained immense popularity for the design of 3D self‐assembled nanostructures. Starting from CNCs, two main routes to synthesize Janus‐type nanorods have been described: 1) polymer grafting and 2) the conjugation of biomolecules.

In their early contribution from 1979 on cellulose‐*g*‐poly(styrene) copolymers, Taga and Inagaki already discovered that the cellulose REG can serve as a polymer grafting site.^[146]^ Later on, REG‐specific polymer grafting on celluloses was realized either by linking the ready‐made polymer to cellulose (grafting‐to) or by attaching a radical initiator to the REGs enabling surface‐initiated polymerization (grafting‐from). In both cases, the REG‐specific grafting requires one of the aldehyde‐specific mechanisms discussed in Section 4. Starting from CNCs, Sipahi‐Sağlam et al. have used both grafting routes.^[77]^ Their grafting‐to protocol involved first the activation of the REGs by a reaction with 4‐hydrazinobezoic acid, followed by attachment of amino‐terminated poly(ethyleneglycol) chains catalyzed by EDC. Grafting‐from was realized via the immobilization of an azo initiator at the REGs that later enabled a topochemical free radical polymerization (FRP).

Radical polymerization techniques have been widely applied to uniformly graft polymers from the CNC surface, including FRP and surface‐initiated atom transfer radical polymerization (SI‐ATRP).[^19^, ^147^] ATRP is a controlled radical polymerization (CRP) method with striking advantages over other techniques, such as functional group tolerance, and control of molar mass and polymer chain architecture. Under ideal conditions, ATRP allows for building the polymer chain in a linear, time‐controlled fashion.^[148]^

**Figure 8:**
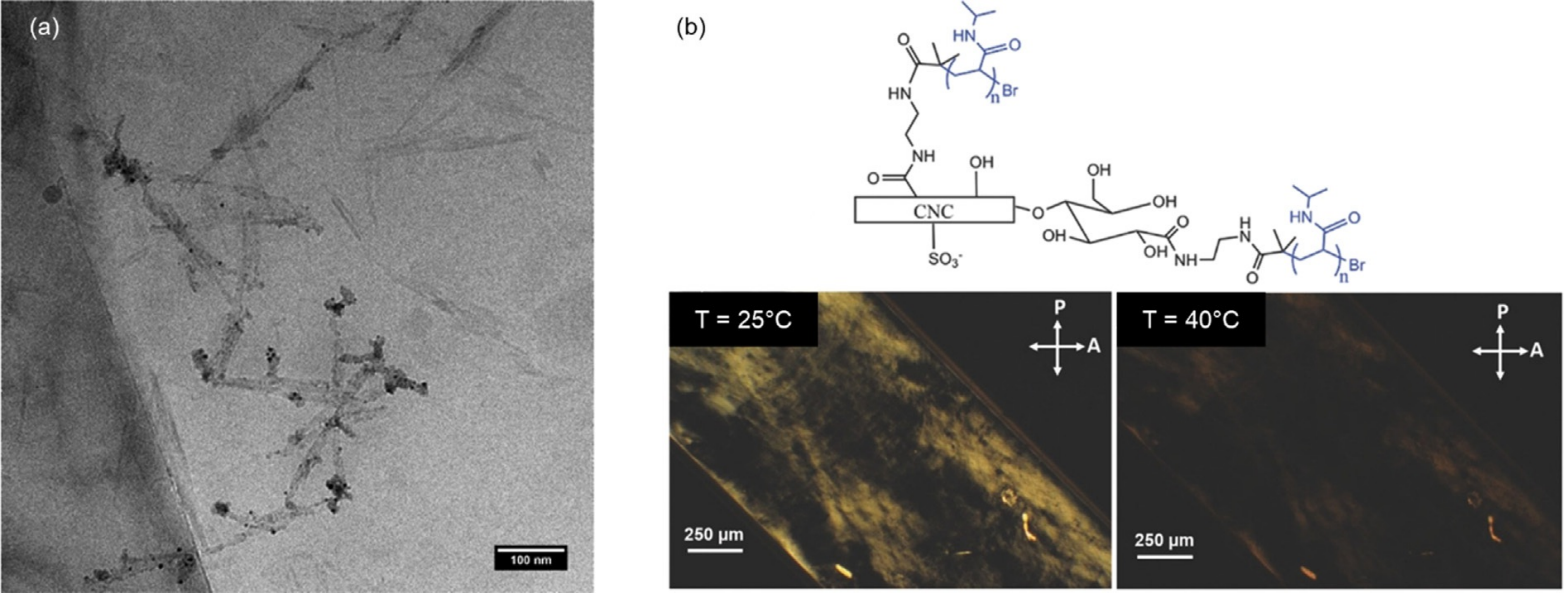
a) Cryo‐EM micrograph of patchy PMETAC‐grafted CNCs labelled with Au^0^NPs.^[103]^ Reproduced with permission, Copyright ACS (2017). b) Polarized optical microscopy images of an aqueous suspension of 10 wt % patchy PNIPAM‐grafted CNCs (schematic structure above) in a glass capillary. Birefringence (LC phase at 25 °C) of the modified CNCs disappears upon heating above the LCST of PNIPAM.^[104]^ Modified with permission from Wiley‐VCH (2017).

Zoppe et al. developed a versatile, water‐tolerant protocol to graft polymers from initiator‐modified CNC REGs via SI‐ATRP and applied this framework to three different polymers (thermoresponsive poly(*N*‐isopropylacrylamide) (PNIPAM), cationic poly([(2‐methacryloyloxy)ethyl]trimethylammonium chloride) (PMETAC), and anionic poly(sodium 4‐vinylbenzenesulfonate) (PSSNa)).^[103]^ They initially oxidized the REGs with NaClO_2_ followed by carbodiimide‐catalyzed amidation in water to selectively conjugate ATRP initiators to the CNC REGs. The modification, however, was not strictly limited to the REGs, resulting in an unselective distribution of the polymer chains on the nanocrystal surface, as visualized by Cryo‐EM and Au^0^NP labelling (Figure 8 a). This “patchy” distribution may be associated with surface impurities or oxidized sites having additional carbonyl and/or carboxyl groups, which again emphasizes the importance of the CNC surface chemistry for the reaction selectivity (Section 4).

In their follow‐up work from 2018, Zoppe and co‐workers investigated the temperature‐dependent, reversible self‐assembly and liquid‐crystalline properties of patchy CNC‐PNIPAM grafts.^[104]^ By polarized optical microscopy (Figure 8 b) they showed that these CNC grafts formed LC phases below the LCST of PNIPAM, which reversibly disappeared upon heating above the LCST, indicating the transition to an isotropic phase. Interestingly, this reversible switching‐on‐and‐off of birefringence was not observed for uniformly grafted PNIPAM CNCs. Based on their findings, the authors foresaw an application of patchy CNC‐polymer grafts as LC temperature switches, e.g., in sensors or smart packaging materials. Moreover, these findings demonstrate that, with respect to the nature of the grafted polymer, end‐wise grafting can significantly affect the stabilization of the CNCs in cholesteric LC phases in aqueous media.^[104]^ This may be translated to a steric stabilization of REG‐functionalized CNCs in nonaqueous liquid conditions when grafted, e.g., with organo‐soluble poly(styrene).

**Figure 9:**
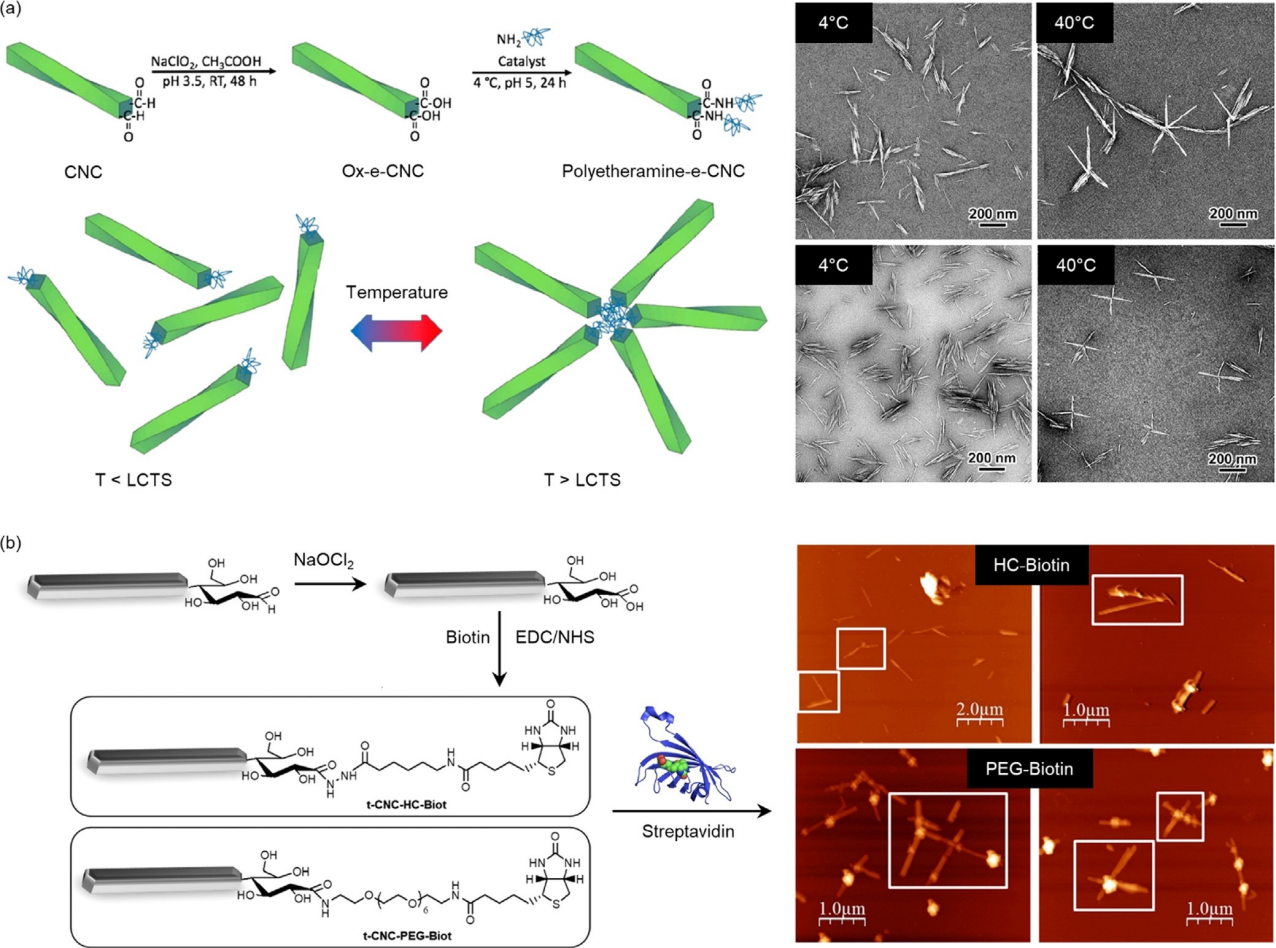
a) End‐functionalization of CNCs with polyetheramines and TEM images showing the temperature‐triggered formation of star‐shaped assemblies above the LCST of the polyetheramine chains.^[22]^ Reproduced with permission, Copyright ACS (2019). b) Tunicate CNCs end‐modified with (+)‐biotinamidohexanoic acid hydrazide (top) or biotin‐dPEG7‐NH_2_ (bottom). The spacer length determines the formation of four‐membered, star‐shaped assemblies with streptavidin (AFM images).^[105]^ Reproduced with permission, Copyright ACS (2018).

Unequivocal, yet qualitative, evidence for the end‐specific modification and Janus‐type behavior of the end‐tethered CNCs was presented by Lin et al.^[22]^ Following a two‐step pathway that mirrored the approach of Arcot et al. (end‐group carboxylation and amide formation),^[88]^ they attached two different types of thermo‐responsive Jeffamine® polyetheramines with known LCSTs to the CNC REGs. This resulted in reversible, star‐shaped assemblies above the LCST of the polyetheramines (Figure 9 a), as demonstrated by TEM imaging and small‐angle neutron scattering (SANS). SANS was used to investigate the particle assemblies above the LCST and the extended chain conformers of the polyethers below the LCST. The results were supported by dynamic light scattering (DLS), showing the reversible change in hydrodynamic diameter upon cycling reversibly (with some hysteresis) from below to above the LCST. Both methods are based on changes in the physico‐chemical behavior of the end‐modified CNCs—an indicative, yet indirect, confirmation of the end‐specific modification.

Similar star‐shaped supramolecular complexes were described by Villares et al. following a different approach that exploits biotin–streptavidin binding (Figure 9 b).^[105]^ Biotin residues with varying spacer length were linked topochemically to tunicate CNCs (t‐CNCs), either in the form of a reactive hydrazide ((+)‐biotinamidohexanoic acid hydrazide (HC‐Biot)) or as a NH_2_‐terminated compound (biotin‐dPEG_7_‐NH_2_ (PEG‐Biot)). The latter protocol followed a sequential acid chlorite oxidation and EDC/NHS amidation. For the PEG‐grafted biotin linker, the binding to streptavidin, bearing four binding sites, led to regular, star‐shaped t‐CNC‐*g*‐biotin–streptavidin assemblies (Figure 9 b). However, this geometry was not observed for the shorter HC‐Biot linker, suggesting a decisive role of the spacer length. Binding studies with QCM‐D on streptavidin‐coated gold surfaces supported this assumption. Accordingly, perpendicular binding of biotinylated t‐CNCs to the protein‐coated gold surfaces was restricted to a minimum length of the spacer (i.e., an oligoethylene glycol chain with seven repeating units), but was also affected by the inherent characteristics of the t‐CNCs (e.g., charge, aspect ratio).

The overall concept behind attaching biomolecules or (bio)active sites to cellulosic surfaces has been widely described, envisaging the design of biointerfaces, drug‐delivery systems, and 3D biohybrid nanostructures.[^149^, ^150^] Targeting the reducing end of CNCs, Karaaslan et al. aimed at asymmetric hybrid polysaccharide–protein nanoparticles, showing directed self‐assembly, via the linkage of a micelle‐forming protein (β‐casein) to azide‐modified CNCs through a Cu‐catalyzed azide‐alkyne click reaction.^[79]^ What they observed, however, were assemblies in different arrangements, without direct confirmation that the protein conjugation was restricted to the REGs.

An example of the application of bioactive, end‐group‐modified CNCs was published by Imlimthan et al., who evaluated their potential for the targeted delivery of diagnostics and therapeutics in vitro and in vivo.^[109]^ The authors developed nuclear imaging probes by labelling the REGs with a hydrazine‐modified diagnostic radionuclide (DOTA=1,4,7,10‐tetraazacyclododecane‐1,4,7,10‐tetraacetic acid), which displayed good biocompatibility in the cell models. In a later study, the DOTA labeling was complemented with a fluorescent dye (cyanine 5), enabling systematic investigations of the nanoparticle biodistribution in vivo by nuclear and optical imaging.^[151]^

### 6.3 Nanocomposites

CNFs and CNCs have both been extensively explored as high‐performance, bio‐based reinforcements for polymer composites.^[152]^ A critical point for the technological exploitation of such nanocomposites, however, is their industrial manufacturability, which hinges in many cases on the limited dispersibility of the CNCs in a polymer of interest, especially if the CNCs are provided in dried form.^[153]^ Similar to oil–water interfaces in emulsions, modifications of the nanocrystal surface with groups serving to compatibilize the nanofiller with the polymer matrix have led to significant improvements in terms of CNC distribution, dispersion stability, and cellulose–polymer interface interactions, which can all lead to an improved mechanical performance of the nanocomposite.[^154^, ^155^] However, in many cases, the reinforcement achieved with surface‐modified CNCs is lower than expected, likely because of the interactions among the CNCs and between the CNCs and the matrix, and thereby pathways for stress‐transfer are weakened.^[156]^ Similar results were observed with physically adsorbed compatibilizers. For example, Dufresne et al. showed that the dispersion of CNCs in polyethylene can be greatly improved by adsorbing poly(ethylene oxide) on the CNC surface (PEO‐CNCs) before blending the PEO‐CNCs with the polymer.^[157]^ The mechanical characteristics of these nanocomposites, however, were hardly improved. On the other hand, Meesorn et al. demonstrated that adsorbing a small amount of amphiphilic poly(vinyl alcohol) onto CNCs aids their dispersion in different matrices and leads to a superior enhancement of the mechanical performance, arguably because hydrogen‐bonding interactions are maintained.[^149^, ^150^, ^151^, ^158^] CNCs that are only partially decorated with a (covalently attached) compatibilizer may offer similar physico‐chemical features, i.e., an increased dispersibility vis‐à‐vis unmodified CNCs and an unmitigated ability to interact with their environment through an abundance of hydrogen bonds.

**Figure 10:**
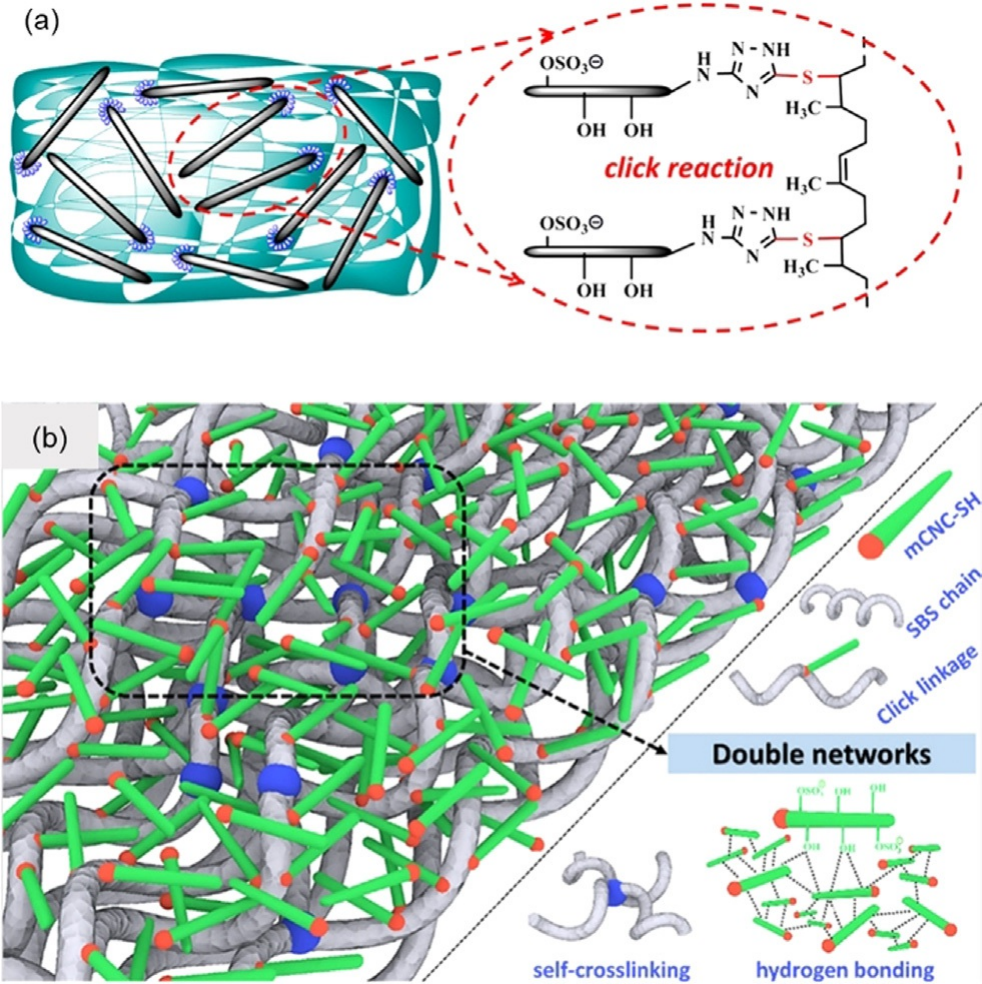
a) Triazole‐end‐grafted CNCs and the proposed covalent conjugation to a natural rubber matrix.^[89]^ Reproduced with permission, Copyright ACS (2018). b) Proposed double‐network structure formed in SBS‐thiol‐CNC nanocomposites.^[90]^ Reproduced with permission, Copyright ACS (2019).

Along these lines, Li et al. investigated the covalent incorporation of CNCs, whose ends had been modified with 3‐amino‐1,2,4‐triazole‐5‐thiol by reductive amination, into a vinyl‐group‐containing rubber matrix via a UV‐induced thiol–ene click reaction (Figure 10 a).^[89]^ The mechanical properties of the nanocomposite were found to gradually increase with the content of end‐functionalized CNCs (2.5–15 wt %) and exceeded the reinforcement achieved with unmodified CNCs. For instance, the storage modulus *E′* (at 25 °C) increased from 1 MPa for the unmodified rubber to 12 MPa upon incorporation of 10 wt % end‐modified CNCs. Compared to that, the aggregation tendency of unmodified CNCs (especially at loadings >7.5 wt %) resulted in weaker composites. While the covalent integration of end‐functionalized CNCs clearly led to improved reinforcement, a comparison with the results achieved with nanocomposites made with poly(vinyl alcohol)‐adsorbed CNCs[^158^, ^159^, ^160^] suggests that there is further room for improvement. A follow‐up study by Lin's group was published very recently, in which the CNC REGs were modified with cysteamine (NH_2_CH_2_SH) via a reductive amination.^[90]^ The selective thiolation of the cellulose REGs was used to covalently integrate the end‐modified CNCs into a thermoplastic elastomer (styrene–butadiene–styrene copolymer (SBS)) via a UV‐induced thiol–ene click during the liquid compounding. Besides enabling covalent nanofiller incorporation, the objective behind the end‐specific modification was to preserve the nanocrystals’ surface properties, ensuring the formation of a stable percolation network with strong filler–matrix and filler–filler interactions. The subsequent UV post‐curing during the molding process formed a double‐network due to the chemical self‐crosslinking of the bulk SBS (Figure 10 b). The results of this approach are promising: the REG‐thiolated CNCs displayed an improved compatibility with the polymer matrix and the nanocomposites exhibited a significantly enhanced mechanical performance compared to reference materials containing unmodified CNCs (e.g., *E′* (25 °C) increased by 40 % to 34 MPa for composites containing 10 wt % thiolated CNCs). It would be interesting to compare these results with nanocomposites made with CNCs that are homogeneously covered with such reactive groups. Moreover, it would appear equally intriguing to utilize amphiphilic Janus CNCs, i.e., CNCs that are end‐functionalized with a hydrophobic polymer, as reinforcing nanofillers in block copolymers featuring blocks of matching polarities or as compatibilizers in otherwise immiscible blends. In both cases, one might expect interesting morphologies and reinforcement effects that take full advantage of the Janus architecture.

### 6.4 Hairy Cellulose Nanocrystals

**Figure 11:**
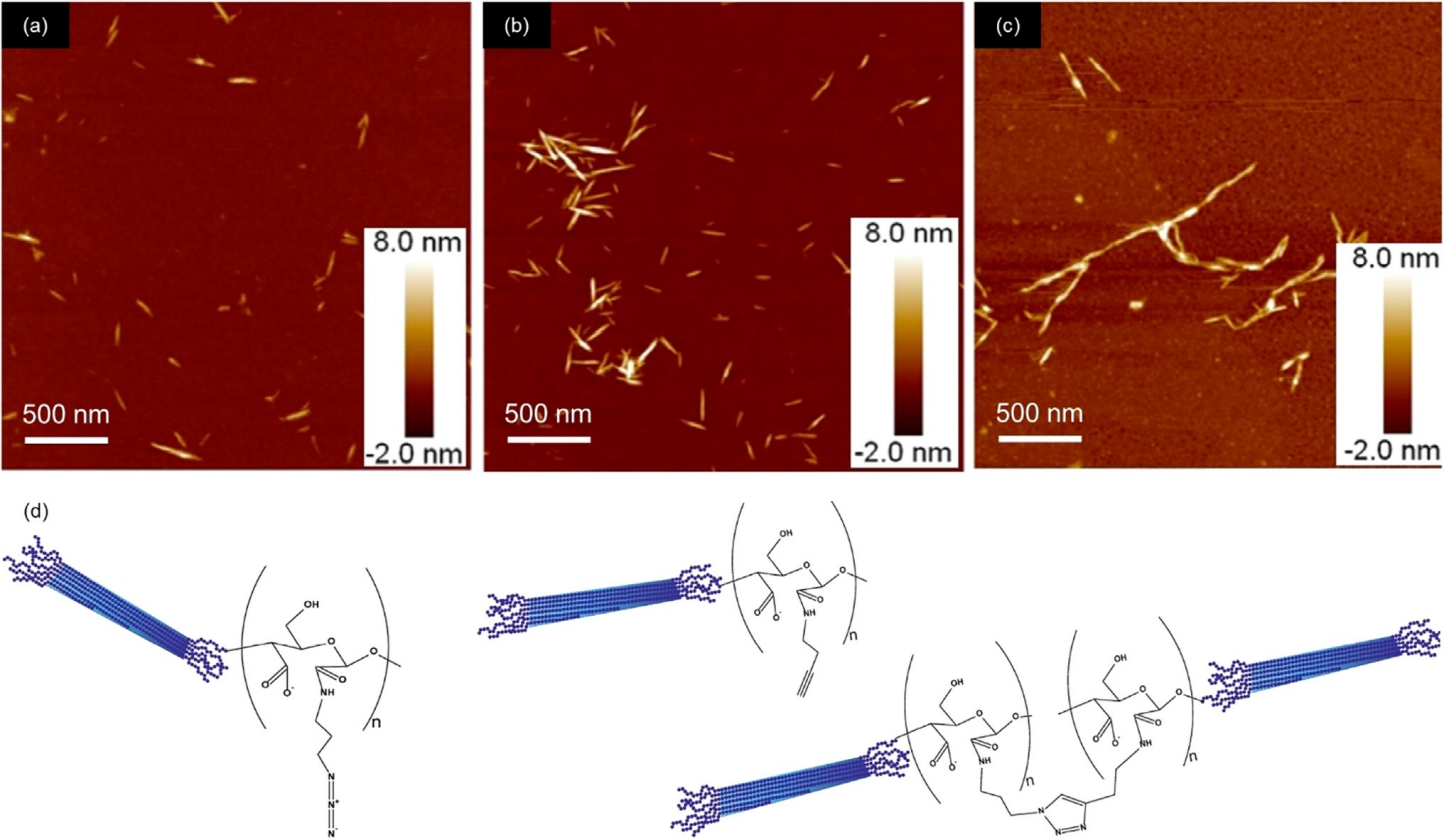
AFM images of hairy cellulose nanocrystals (hCNCs) (a,b), and end‐to‐end connected hCNCs (c) following the bottom‐up route illustrated below (azide–alkyne click reaction between end‐modified hCNCs).^[161]^ Reproduced with permission, Copyright Elsevier (2017).

An entirely different approach toward symmetrically end‐group‐functionalized CNCs was published by van de Ven's group.^[162]^ The basic principle exploited here was the application of a periodate oxidation to cellulose pulps affording CNCs (Figure 11 a/b) with dialdehyde groups on both ends.[^163^, ^164^] Rather than representing a route for chemically modifying REGs, this approach can be seen as an alternative method for CNC isolation. The underlying principle can be related to cellulose crystallinity: dislocations—referred to as amorphous regions—in the cellulose microfibril are kinetically more accessible for the oxidant than the crystalline regions.[^165^, ^166^] Thus, when the fibers react with periodate, C2−C3 bond cleavage in the AGUs and dialdehyde formation occur preferably in disordered fiber regions. Accompanied by heating (80 °C), the cellulose dialdehyde chains were cleaved off, liberating the so‐called hairy cellulose nanocrystalloids (hCNCs) having aldehyde functionalities on both ends (Figure 11 a,b).^[164]^ The “hairy” appearance of these nanocrystals resulted from protruding chains which were still attached at both crystal ends. These chains enhanced the colloidal stabilization of the uncharged hCNCs in water.^[164]^ The high aldehyde content at the hCNC ends makes them particularly interesting for aldehyde‐specific modification, including oxidative treatments,^[163]^ Schiff base reactions,^[167]^ and cationization.^[168]^ This paves the way toward advanced materials applications, ranging from aerogels and packing materials to wastewater treatment.^[162]^ One very interesting example is the bottom‐up route toward end‐to‐end connected, nanofiber‐like hCNCs, which was realized through an end‐functionalization with 1‐amino‐3‐butyne or 3‐azido‐1‐propanamine, enabling a subsequent click reaction (Figure 11 c,d).^[169]^

## 7 Confirmation and Quantification of the End‐wise Modification

**Figure 12:**
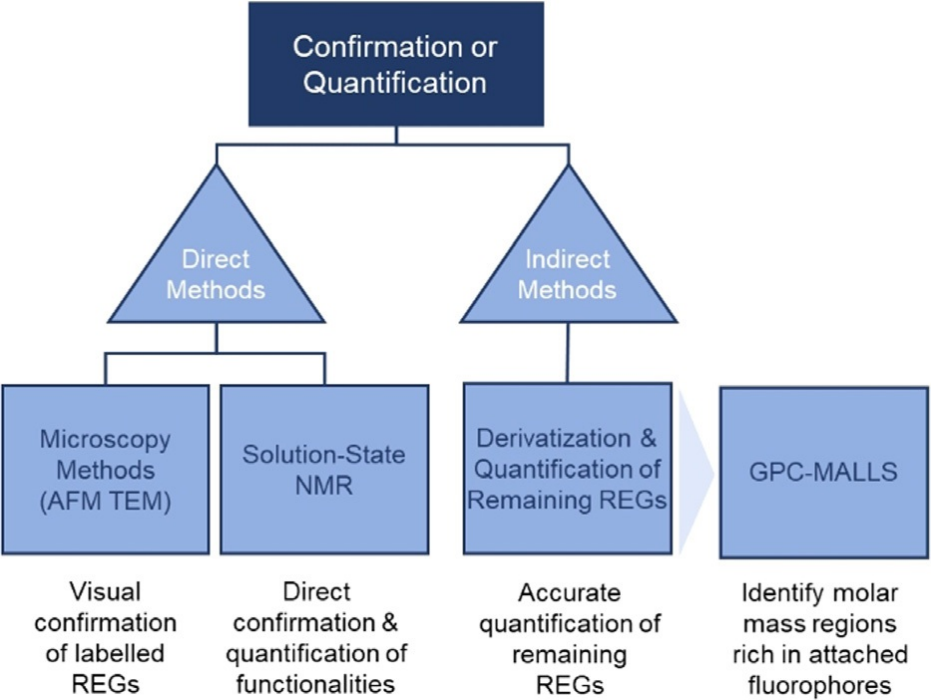
Direct and indirect analytical methods for detection and quantification of cellulose end‐specific modification. (GPC‐MALLS: after REG derivatization with a fluorophore).

The confirmation and quantification of the REG modification is challenging, as the spatial volume and the number of introduced functionalities is typically very low, compared to the bulk AGUs. Common spectroscopic techniques are not usually applicable, as the modification is either close to the detection limit of the instrument or spatial and/or spectral resolution is poor. Similarly, scattering methods are not useful for direct analysis because the dimensional changes due to REG modification are typically minute. The only applicable direct spectroscopic method at the moment is solution‐state NMR spectroscopy (Figure 12). Another way to gain direct evidence for modified REGs is to specifically label them with species that can be made visible by electron microscopy. Indirect methods, in turn, generally require a reaction with the remaining REGs after the modification and quantification of the outcome. It is also possible to track the changes in the physico‐chemical behavior of the modified CNCs (see Section 6). However, these methods, often relying on colloidal self‐assembly, are not dealt with here because of the lack of any quantitative character.

### 7.1 Direct Approaches—Electron Microscopy

To confirm REG modification, functionalities must be introduced that are large and conductive enough to offer sufficient changes in contrast for detection by microscopy, which is generally not provided by low‐molecular‐weight (in)organic compounds. Indeed, attachment of metal nanoparticles can be considered the classical approach to observe REG modifications by microscopy (Figure 2). More recently, Lokanathan et al. introduced Ag^0^NPs to the REGs via reductive amination with a thiol‐terminated primary amine and then a reduction of AgNO_3_ with borohydride.^[87]^

While electron microscopy coupled with labeling may be visually attractive to yield a concrete picture of the phenomenon, it does not provide actual confirmation or accurate quantification of the specific REG linking chemistry.

### 7.2 Direct Approaches—NMR Spectroscopy

Nearly all modern REG modifications discussed here involve the use of CNCs which form a particle suspension, not a molecular solution. Therefore, solution‐state NMR spectroscopy is not directly applicable to CNCs in common molecular solvents. While solid‐state ^13^C cross polarization magic angle spinning (CP MAS) NMR spectroscopy has become very useful in the determination of crystallinity of samples,[^170^, ^171^, ^172^] the resolution and signal‐to‐noise ratio are still not good enough for it to be applied to confirm REG modifications. On the other hand, ^13^C CP MAS calibration (against other quantitative methods) may in the future offer a convenient and relatively rapid technique for monitoring REG modification, if robust chemistry is already established.

**Figure 13:**
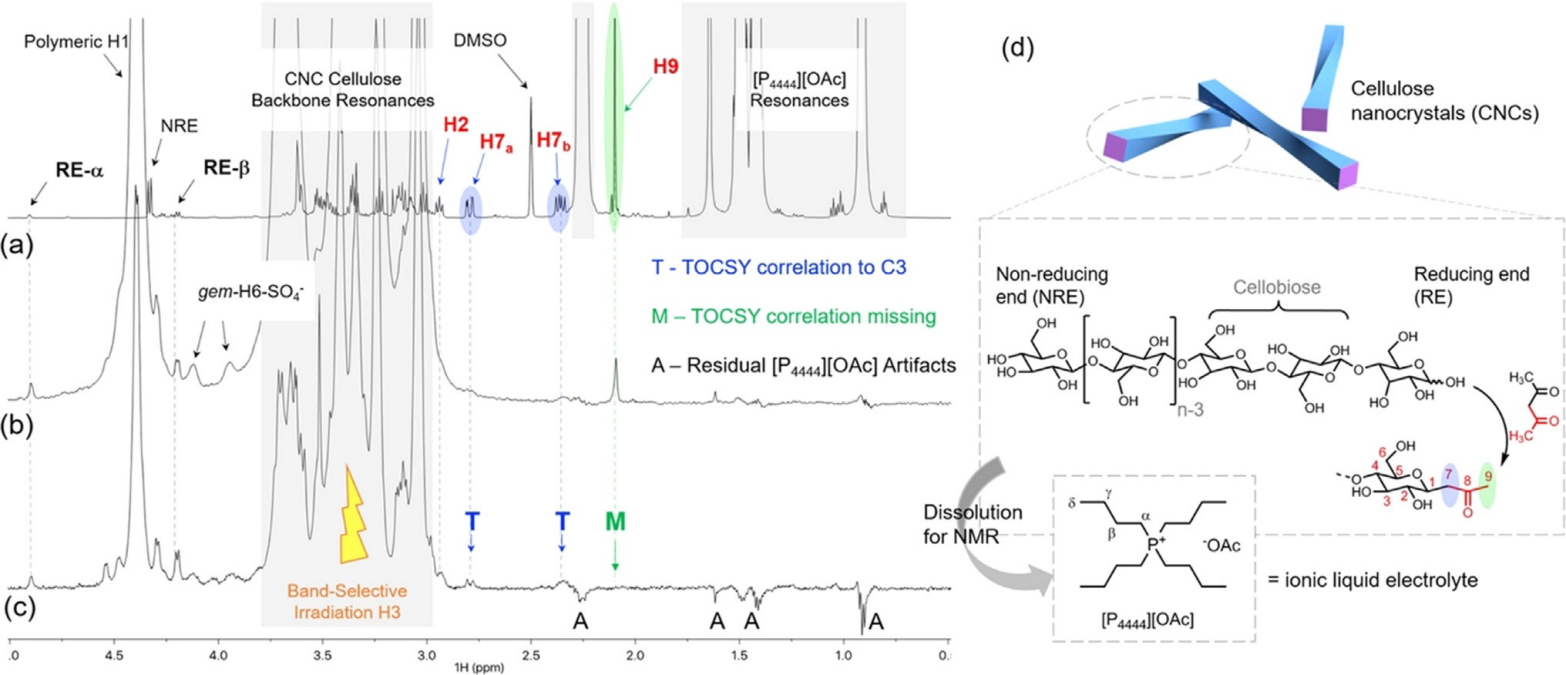
Identification of the Knoevenagel condensation reaction product on CNCs using solution‐state NMR spectroscopy in the [P_4444_][OAc]/[D_6_]DMSO electrolyte at 65 °C: a) ^1^H spectrum of the condensed cellobiose model compound in [P_4444_][OAc]/[D_6_]DMSO; b) diffusion‐edited ^1^H spectrum of the condensed CNCs showing the presence of key resonances; c) band‐selective TOCSY experiment showing correlations not extending over the introduced carbonyl; d) modification scheme.^[75]^ Reproduced with permission, Copyright ACS (2019).

With much shorter *T*_1_ relaxation rates and longer *T*_2_ values, the pulse repetition is faster and the resolution is higher in solution‐state NMR spectroscopy. However, cellulose is not a trivial substance to dissolve and the required perdeuterated cellulose solvents are scarcely available.^[173]^ Common ionic liquids for cellulose dissolution, such as 1‐ethyl‐3‐methylimidazolium acetate, have the disadvantage that many of their resonance signals overlap with those of the cellulose backbone. Their cellulose solutions are also highly viscous, leading to poor solubility and signal broadening. Dialkylimidazolium acetate ionic liquids, as the most effective and studied ionic liquids for cellulose dissolution, are also known to react with cellulose reducing ends.[^174^, ^175^] Fortunately, tetraalkylphosphonium acetate salts, in the form of their [D_6_]DMSO electrolytes, are highly effective at cellulose dissolution.^[175]^ They are among the most thermally and chemically stable cation class of ionic liquids and have not yet been determined to react with cellulose REGs. While tetraalkylphosphonium ylides have been isolated, this has only been achieved by reaction of the corresponding halide salts with very strong inorganic bases, such as *n*‐butyllithium. This opens the door to the quantification of the REGs vs. the bulk AGUs if the molar mass is low enough, such that the REG signals are abundant enough to be quantified with any accuracy.[^75^, ^176^] Of the possible tetraalkylphosphonium acetate homologues, tetrabutylphosphonium acetate ([P_4444_][OAc]) is able to dissolve at least 5 wt % cellulose as a 20:80 wt % [P_4444_][OAc]/[D_6_]DMSO composition. At a 5 wt % composition at 65 °C, excellent resolution is afforded to nanocelluloses.[^75^, ^177^, ^178^] Resolution is such that a series of 2D correlation experiments can be recorded, allowing for assignment of cellulose and chemically modified cellulose resonances. *T*_2_ values are often long enough^[177]^ to allow for most solution‐state NMR experiments that are typically used in organic chemistry. One key experiment that sidesteps the issue of overlap of [P_4444_][OAc] resonances in the alkyl frequency region, is the application of diffusion‐editing.^[177]^ This allows for editing out of the fast diffusing species, i.e., [P_4444_][OAc], and retention of the slow‐diffusion species, i.e., polymeric species. Indeed, it has been possible to confirm and quantify REG modification introduced by Knoevenagel condensation (Section 4.4) by using this technique.^[75]^ The reaction was also performed on cellobiose to prepare a model compound for NMR signal assignment. Key resonances of the condensation product were overlapping with the [P_4444_][OAc] signals, so diffusion‐editing was applied to the modified CNC product, enabling confirmation and rough quantification of the reaction (Figure 13). Through deconvolution of the signals of the introduced carbonyl methyl groups for the ^1^H NMR and diffusion‐edited ^1^H NMR spectra vs. the cellulose backbone signals, it was possible to arrive at reasonably accurate REG conversion values.

Currently, one disadvantage of liquid‐state NMR in REG modification analysis is the extra work that goes into resonance assignment to meet the current standards required by organic chemists. However, if introduced functionalities are relatively simple (e.g., methyl groups below 3 ppm in the ^1^H NMR spectrum) diffusion‐editing is a very rapid experiment to confirm the reaction and even give rough quantification measures, with spectral deconvolution. In the long run, the application of cellobiose as a REG model has considerable value, as the regioselectivity of the reaction may be determined with more confidence. Another drawback is the lack of a commercially available electrolyte solution for research. However, this will likely change as the method starts to mature.

In cases where the degree of substitution (DS) of some functionality is high enough, the cellulose sample may be soluble in common perdeuterated molecular solvents. This requires a derivatization step that sufficiently disrupts the crystalline structure of cellulose and should therefore not be considered as a direct analytical method. However, it is worth looking at one specific case where cellulose samples have been peracetylated. The REGs resonances were identified and a selective deacetylation of the REG “anomeric” acetate was demonstrated.^[179]^ Different cellulose samples—cotton, sulfite pulp, and regenerated celluloses—were hydrolyzed to their leveling‐off degrees of polymerization (LODP, DP_N_=23–68) and subsequently peracetylated. Band‐selective TOCSY and other 2D correlation techniques were used to identify the REG spin systems. The samples were then treated with benzylamine to selectively deacetylate the REG anomeric acetate. Because the method allows for solvation of the polymer to a high degree, resulting in enhanced resolution, it may have value in improved quantification of REGs.

### 7.3 Indirect Approaches—Derivatization and Quantification of Remaining REGs

The term “reducing sugars”, and hence REG, comes from the fact that some metal ions are reduced in their presence, forming the basis of several laboratory tests used to identify reducing sugars. For example, Benedict's test involves the reduction of CuSO_4_ (Cu^II^, blue solution) to Cu_2_O (Cu^I^, red precipitate). However, more modern methods have evolved to quantify REGs in cellulose and nanocelluloses. Some of these are based on photometric quantification of reduced metal species or potentiometric titration of formed acid after REG reaction. Other methods are based on the attachment of labeled REG compounds, which absorb or fluoresce in the visible or UV region, for direct photometric quantification. Thus, specific methods have been assessed to a certain degree for the quantification of REGs but also for the remaining REGs after certain modification reactions.

**Figure 14:**
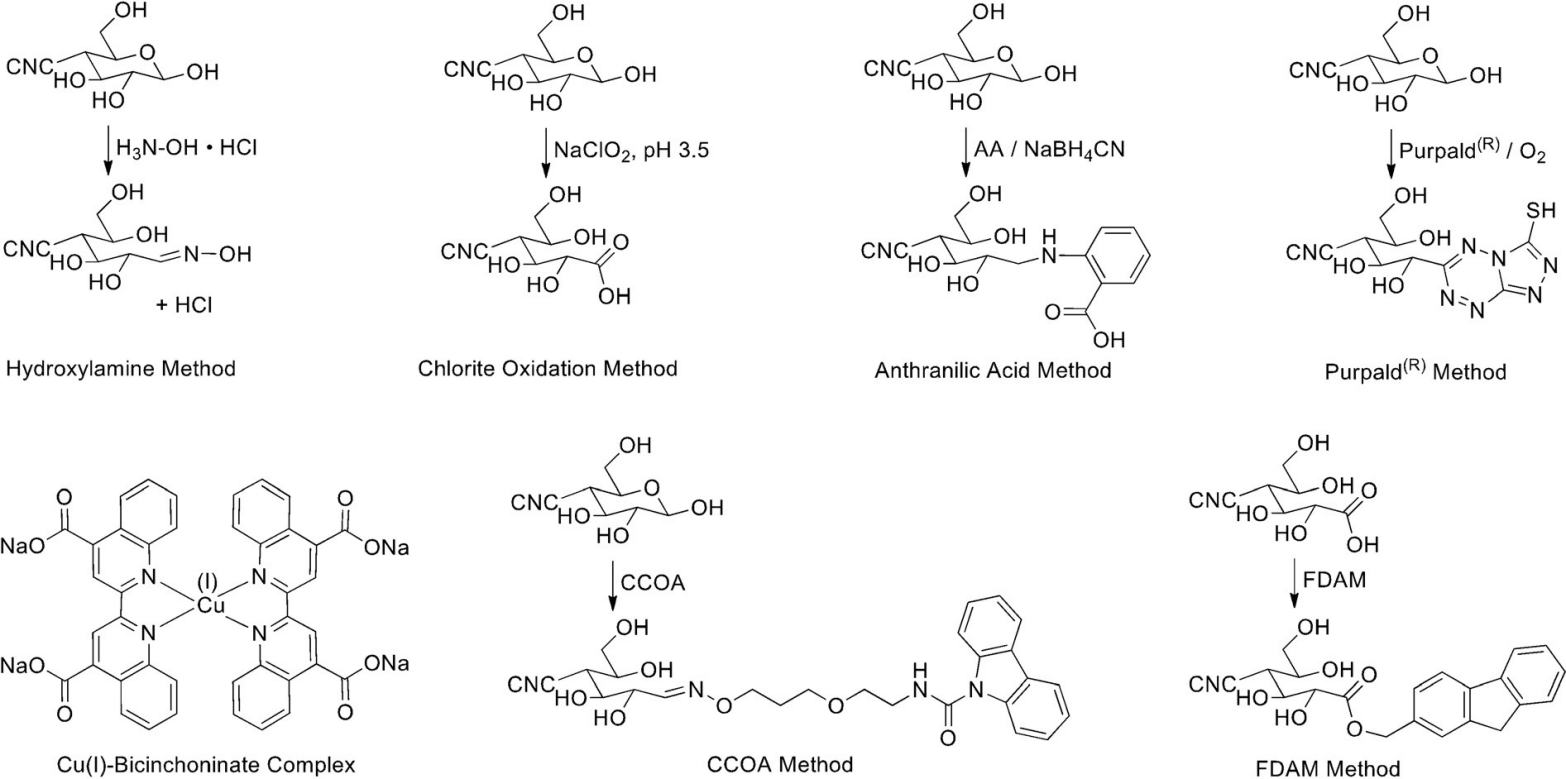
Derivatization methods for the quantification of REGs.

Despite the low abundance of REGs, titration methods have been used and give values close to those expected for CNCs. Tang et al. quantified REGs using two different chemical methods, followed by potentiometric titration.^[110]^ The first method was the classical “hydroxylamine method” by reaction of REG aldehydes with hydroxylamine hydrochloride, forming oximes (Figure 14) and one mole of HCl per REG. The generated HCl was then titrated with NaOH to yield a REG value of 24.5 μmol g^−1^ (DP_N_=252) for CNCs derived from the H_2_SO_4_ hydrolysis of bleached softwood kraft pulp. The second method required acid chlorite oxidation of the REGs to yield carboxylic acids (Figure 14). The acids were then conductometrically titrated with NaOH to quantify the number of oxidized REGs. This method gave a REG value of 31.1 μmol g^−1^ (DP_N_=198) for the same CNCs. The more efficient hydroxylamine method was then applied for quantifying the remaining REGs after grafting amine‐terminated poly(styrene) to the REGs, which showed that the aldehyde content decreased from 24.5 to 11.4 μmol g^−1^.

Risteen et al.^[104]^ and Velleste et al.^[56]^ have applied the “2,2′‐bicinchoninate (BCA) method”,^[180]^ based on reduction of Cu^II^ to Cu^I^, which is quantified spectrophotometrically as the Cu^I^ bicinchoninate ([Cu^I^(BCA)_2_]^3−^) complex (Figure 14) at 560 nm. Risteen et al. determined a value of 18.2 μmol g^−1^ of REGs (DP_N_=339) for commercial CNCs from the USDA Forest Service, Forest Products Laboratory (Table S1), derived from bleached softwood dissolving pulp.^[104]^ Velleste et al. determined a value of 29.7 μmol g^−1^ of REGs (DP_N_=208) for Avicel PH‐101 (50 μm, Fluka) microcrystalline cellulose (MCC) which had been regenerated from phosphoric acid.^[56]^ Bacterial nanocellulose (BCN), derived from Nata de Coco (CHAOKOH coconut gel in syrup, Theppadungporn Coconut Co., Ltd, Thailand), was also analyzed, giving a value of 2.8 μmol g^−1^ of REGs (DP_N_=2205).

Velleste et al. also used a comparative labelling method, the “anthranilic acid (AA) method”, for the direct labelling of REGs (Figure 14) under reductive amination conditions.^[56]^ In this case, they labelled the REGs directly, prior to a fluorescence emission (at 330 and 425 nm) measurement of the labelled compounds. As such, they employed a high‐dose cellulase treatment (20 FPU g^−1^ *T. reesei* cellulase at 25 °C overnight) to render the insoluble samples soluble for solution‐state quantification after derivatization and centrifugation. The BCN was also analyzed using this method, giving a value of 4.9 μmol g^−1^ of REGs (DP_N_=1260). A range of other pulp‐ and cotton‐based substrates were analyzed by both methods and the ratios gave an average overestimation of 1.43 in favor of the AA method when higher molar mass (MM) celluloses were considered. However, the lower molar mass MCC gave a discrepancy of 1.2, in favor of the AA method.

Another method, based on direct labelling and quantification is the “Purpald® method”,^[182]^ which relies on conjugation of aldehydes with 4‐amino‐3‐hydrazino‐5‐mercapto‐1,2,4‐triazole (Purpald®) to yield an intermediate that can be quantified by spectrophotometry (570 nm) after oxidation with a mild oxidant. Criado et al. used this method to quantify REGs in commercial CNCs, derived from H_2_SO_4_ hydrolysis of bleached softwood kraft pulp (FPInnovations’ Pilot Plant, Canada).^[183]^ They determined a value of 20±4 μmol g^−1^ of REGs (DP_N_=309±62). The method was further used to quantify the increasing aldehyde content upon gamma‐irradiation of the CNC suspensions, to better understand their antioxidant properties.

**Figure 15:**
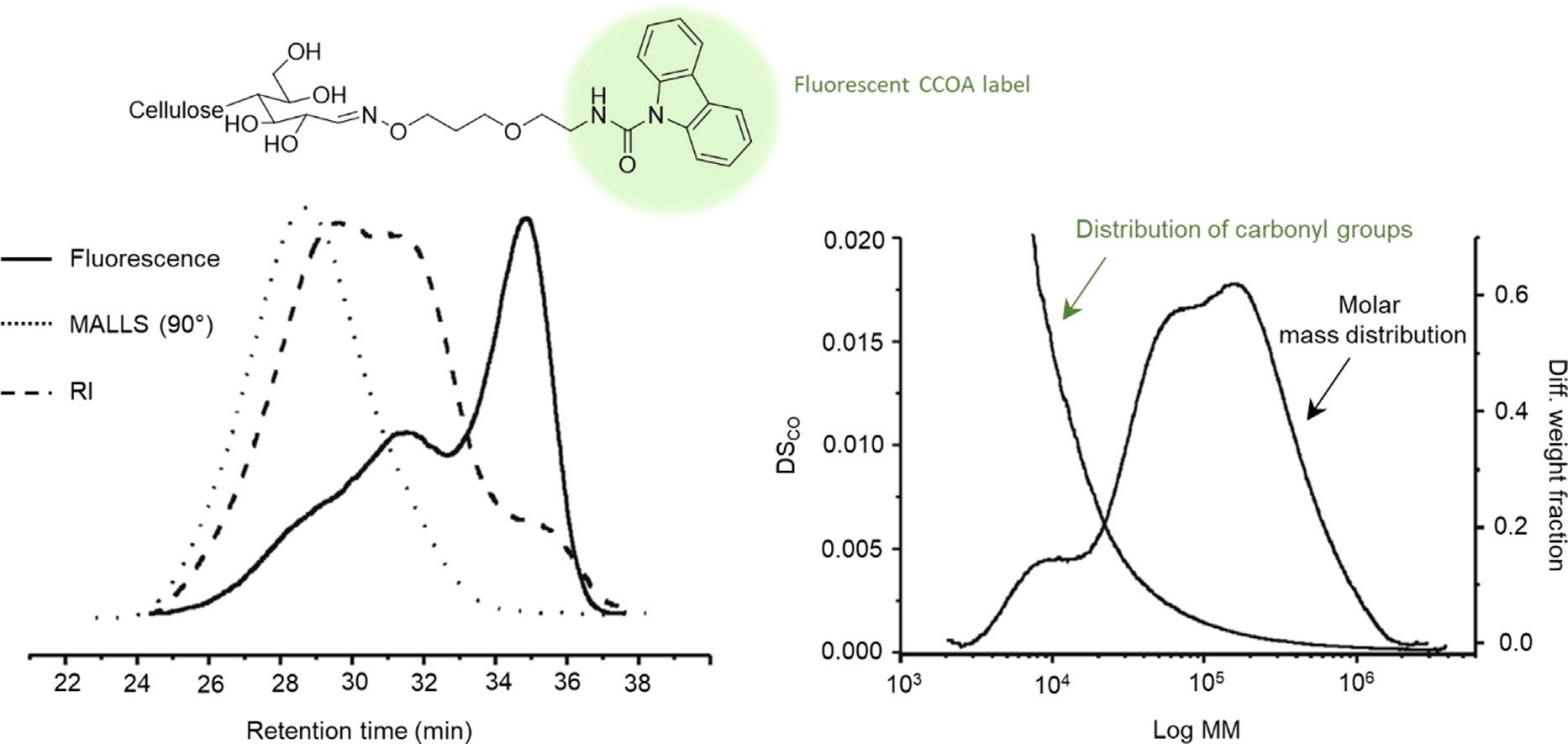
Combination of GPC with fluorescence and MALLS detection for quantifying REGs and REG distribution in cellulosic substrates, as a function of the molar mass (MM) after labeling with CCOA; left: GPC traces with RI, fluorescence, and MALLs detection; right: differential weight fraction and degree of substitution for REGs (DS_CO_), as a function of the MM.^[181]^ Modified with permission from Springer‐Verlag (2006).

Meanwhile, Heise et al. utilized the carbazole‐9‐carbonyl‐oxyamine (CCOA) method[^73^, ^74^] to quantify the REGs in pristine CNCs from H_2_SO_4_ hydrolysis of cotton linters.^[75]^ The value of 84.0 μmol g^−1^ of REGs (DP_N_=74) determined was comparable to the value of 95.1 μmol g^−1^ of REGs (DP_N_=65), determined by solution‐state NMR spectroscopy (Section 7.2). The method involves a reaction of a fluorescent‐labelled alkoxyamine (Figure 14). The chemistry itself is well‐studied and determined to be stoichiometric for REG conversion, given sufficient time and suitable conditions for the reaction (Table 1). The real advantage of the method is that the labelled REGs are stable in DMAc/LiCl, enabling GPC measurements for determination of MM distributions. The use of the fluorescent label also allows for fluorescence, RI, and MALLS detection, where the abundance of REGs, as a function of the MM, can be accurately determined (Figure 15).^[181]^ This provides relevant information, as large fractions of the REGs can be expected in the low‐MM region, which can be associated with the size distribution of the CNCs. While this method has so far only been applied to quantify REGs in pristine CNCs, there is significant potential for quantifying residual REGs after REG modification reactions. One may also consider more selective pathways, targeting surface oxidation points vs. REGs, as the CCOA method also converts ketones to the corresponding oxime products.

Finally, in a method similar to CCOA, the “9*H*‐fluoren‐2‐yl‐diazomethane (FDAM) method” can be used to quantify the carboxylic acids in cellulose.^[184]^ The chemistry is based on reaction of carboxylic acids with the diazo‐modified fluorophore in DMAc to form stable esters. While this has not been directly applied to nanocellulose, it offers the same potential for the determination of carboxylic acid contents, as a function of the MM, when combined with RI, MALLS, and fluorescence detection. This may be applied in cases where the REGs require oxidation to carboxylic acids, prior to further modification. Alternatively, REGs may be oxidized using acid chlorite oxidation. This method might also allow for determination of carboxylic acids along the nanocellulose surfaces, which is not uncommon for several CNC or CNF preparation methods. A current disadvantage, however, of the CCOA and FDAM methods is that both cases the reagents are not yet commercially available and must, therefore, be prepared following the established protocols.

### 7.4 Method Comparison and Conclusions

A direct comparison of the methods clearly shows that a combination of complimentary analysis should always be employed when confirming, quantifying, and assessing the applicability of any REG modification method (Table 2). So far, the only direct method with sufficient resolution to discriminate between REG chemical species is solution‐state NMR spectroscopy in an ionic liquid electrolyte.[^75^, ^177^] While the workflow required to validate this approach for each new functionality can be tedious, the value in applying this method in the long run is likely considerable. As solution‐state NMR analysis is standard practice for characterizing newly synthesized organic molecules, we foresee rapid developments in the method and its associated workflows with ionic liquids. However, indirect methods, especially those for quantifying REGs, should also be applied as they have the potential to yield accurate values.

**Table 2:** Comparison of direct and indirect methods for confirming and quantifying REG modification.

Method	Sample preparation	Detection	Pros/Cons	Ref.
AFM	drying on surface	microscopy	− requires introduction of bulky functionality + direct confirmation of reaction	[105]
				
TEM	staining & drying on TEM grids or setting in polymer gel for cutting (Cryo‐EM: freezing in holey TEM grids)	microscopy	− requires introduction of bulky functionality, with sufficient contrast + direct confirmation of reaction + can be combined with electron diffraction	[22, 54, 88, 103]
				
solution‐state NMR	dissolution into [P_4444_][OAc]/[D_6_]DMSO	NMR	+ high‐resolution quantitative information on chemical regioselectivity is possible − interpretation can be complicated and may require model compound synthesis	[75, 176, 177]
				
BCA	formation of Cu^I^ bicinchoninate complex after Cu^II^ oxidation	photometric absorption at 560 nm	+ simple method for REG quantification − further validation studies required for CNCs	[56, 104]
				
AA	reductive amination with anthranilic acid	fluorescence emission at 330 & 425 nm	+ simple method for REG quantification − further validation studies required for CNCs	[56]
				
hydroxylamine	formation of oxime and HCl by reaction with hydroxylamine hydrochloride	pH titration with NaOH	+ simple method for REG quantification − further validation studies required for CNCs	[110]
				
chlorite oxidation	NaClO_2_ oxidation to carboxylates at pH 3.5 and acidification to carboxylic acids	conductivity titration with NaOH	+ simple method for REG quantification as carboxylates − further validation studies required for CNCs	[110, 184]
				
Purpald®	reaction with Purpald® reagent under oxidative conditions	photometric absorption at 570 nm	+ simple method for REG quantification − further validation studies required for CNCs	[183]
				
CCOA	reaction with CCOA alkoxyamine to form oxime, under heterogeneous (aqueous) or homogeneous (LiCl/DMA) conditions	GPC‐MALLS with fluorescence and RI detection	+ well‐validated method for REG determination + may potentially offer more information than simple REG quantification‐based methods − time‐consuming	[73, 74, 75]
				
FDAM	reaction of carboxylic acids with FDAM diazo compound to form ester (REGs first require chlorite oxidation & acidification)	GPC‐MALLS with fluorescence and RI detection	+ well‐validated method for carboxylic acid determination − time‐consuming + may potentially offer more information than simple REG quantification‐based methods	[184]

## 8 Conclusions and Outlook

Because of the directionality of the cellulose chain, reducing end‐groups (REGs) in the open‐chain aldehyde form may be targeted with topochemical reactions, which also enables end‐wise modification of native nanocellulose objects like CNCs. We see that this Review appears at a watershed where many of the fundamental challenges—such as accessibility, established mechanisms, and characterization—have been at least partially resolved and the first materials applications are budding. Templated assembly, anisotropically responsive structures, and smart composites, with interfacial engineering, are among the concepts that are already within reach. Anisotropic amphiphilic particles, such as Janus rods, engineered by relying on the principles of end‐wise modification, could serve as components in the next‐generation nanomachines. In the bigger picture, such issues are important when renewable, bio‐based nanomaterials are updated for the emerging decade, particularly in the light of the industrial production of nanocellulose—a scenario that has been slowly maturing for the past decade (see the Supporting Information). Instead of seeing bio‐based materials as simply “renewable” or “sustainable”, one can introduce generic, modular concepts within materials science where aspects of their original, biosynthetic structure are utilized.

## Conflict of interest

The authors declare no conflict of interest.

## Biographical Information

*Katja Heise is a postdoctoral researcher at Aalto University (Finland) and works as a part of the FinnCERES community (Aalto University and VTT*, *Finland) in the group of Eero Kontturi. She received her PhD in 2017 from TU Dresden (Germany) under the direction of Prof. S. Fischer. Her current research lies at the interface between nanocellulose materials science and organic synthesis*.

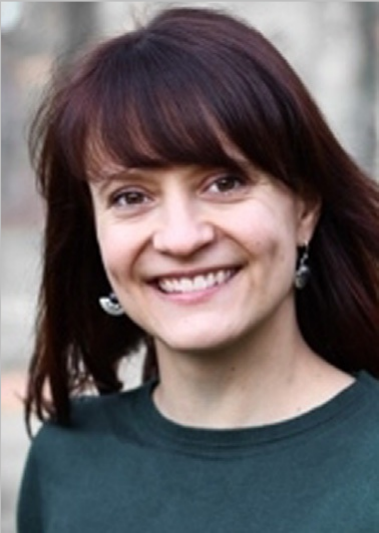

## Biographical Information

*Gwendoline Delepierre has a Master's degree in chemistry from KU Leuven (Belgium), where she was involved in various research projects on cellulose nanocrystals. She started her PhD at the Adolphe Merkle Institute in 2017 under the supervision of Prof. Christoph Weder and Dr. Justin Zoppe. She is currently on an exchange at the University of British Columbia in Vancouver (Canada) under the supervision of Prof. Emily Cranston*.

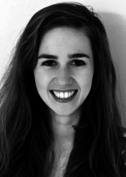

## Biographical Information

*Eero Kontturi earned a PhD in surface science (Supervisor: Prof. J. W. Niemantsverdriet, TU Eindhoven, The Netherlands, 2005), and he gained international experience at Pierre‐and‐Marie‐Curie University (Paris, France), University of Vienna (Austria), and Imperial College London (UK). He was appointed associate professor in Aalto University in 2014. His research focuses on interfacial phenomena with cellulosic materials*.

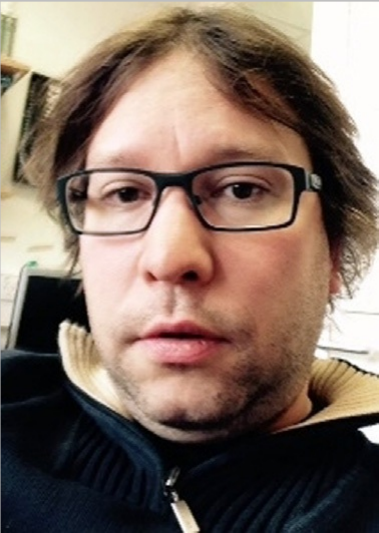

## Supporting information

As a service to our authors and readers, this journal provides supporting information supplied by the authors. Such materials are peer reviewed and may be re‐organized for online delivery, but are not copy‐edited or typeset. Technical support issues arising from supporting information (other than missing files) should be addressed to the authors.

Supplementary
