# Supplementary material for: Chemical Modification of Reducing End‐Groups in Cellulose Nanocrystals
Source: Angew Chem Int Ed Engl. 2020 Sep 9;60(1):66–87. doi: 10.1002/anie.202002433 (PMC7821002; doi:10.1002/anie.202002433)
Supplement: Supplementary file 1 — Supplementary [file ANIE-60-66-s001.pdf]

Angewandte Chemie
Eine Zeitschrift der Gesellschaft Deutscher Chemiker

Supporting Information

Chemical Modification of Reducing End-Groups in Cellulose Nanocrystals

Katja Heise[+], Gwendoline Delepierre[+], Alistair W. T. King, Mauri A. Kostiainen, Justin Zoppe, Christoph Weder, and Eero Kontturi**

anie_202002433_sm_miscellaneous_information.pdf

SUPPORTING INFORMATION

Table of Contents

	Page
S1. Commercially available cellulose nanocrystals	1
S2. Cellulose nanocrystals used for end-wise modification	2
References	3

S1. Commercially available cellulose nanocrystals

Table S1 Industrially produced CNCs, their biomass source, the production volume, and the surface chemistry of the CNCs.[1–4]

Company	Biomass source(s)	Production volume	CNC surface Chemistry	References
Alberta-Pacific Forest Industries Inc., Canada	Hard/softwood Kraft pulp and dissolving pulp	500 kg/day	Sulfated	[5]
Anomera Inc., Canada	Softwood pulp	30 kg/day (current); 1,000 kg/day (projected 2020)	Carboxylated	[6]
Blue Goose Biorefineries Inc., Canada	Viscose grade dissolving pulp	10 kg/day	Carboxylated	[7]
CelluForce Inc. (also procured from the University of Maine), Canada	Bleached softwood Kraft pulp	1,000 kg/day (since 2012)	Sulfated	
Cellulose Lab, Canada	Dissolving/commercial pulp, cotton, sisal, tunicate	10 kg/day	Sulfated (+ surface modifications)	
GranBio (formerly American Process Inc.), USA	Woodchips (eucalyptus), agricultural residues, energy crops	500 kg/day	Unmodified (+ grade containing lignin coating)	[8–12]
FPInnovations, Canada	Bleached chemical wood pulp	1.5 kg/day	Sulfated/ phosphorylated	
InnoTech Alberta, Canada	Various bleached hard/softwood pulp	2 kg/day	Sulfated	
Melodea Ltd., Israel	Various bleached hard/softwood pulp	>10,000 kg/year (projected 2020)	Sulfated	
USDA Forest Products Laboratory, USA	Dissolving pulp	10 kg/day	Sulfated	

Market reports on nanocellulose – URL: https://www.researchandmarkets.com/reports/4827614/the-global-market-for-nanocellulose#rela1-4830123

S2. Cellulose nanocrystals used for end-wise modification

Table S2. List of cellulose nanocrystals (commercial and non-commercial) used for end-wise modifications.

Cellulose source	Cellulose/CNC supplier	Isolation approach	CNC surface chemistry	References
Whatman 1 ashless filter paper (cotton linters)	Cellulose: Whatman GmbH (Dassel, Germany)	H_2SO_4 hydrolysis (64 wt% H_2SO_4, 45 min, 45 °C)	Sulfated	[13–16]
Whatman 1 ashless filter paper (cotton linters)	Cellulose: Whatman GmbH (Dassel, Germany)	HBr hydrolysis (1.5-4 M HBr, 1-4 h, 100 °C)	Uncharged	[17]
Whatman 1 ashless filter paper (cotton linters)	Cellulose: Whatman GmbH (Dassel, Germany)	HCl hydrolysis (2.5 M HCl, 30 min, 90 °C)	Uncharged	[18]
Softwood sulfite pulp	Cellulose: Xinhua Paper Mill (Hangzhou, China)	H_2SO_4 hydrolysis (64 wt% H_2SO_4, 45 min, 90 °C)	Sulfated	[19]
Cotton linters	-	(i) NaOH pretreatment (2 wt%, 12 h)<br>(ii) H_2SO_4 hydrolysis (64 wt%, 1 h, 45 °C)	Sulfated	[20]
Cotton linters	-	H_2SO_4 hydrolysis (64-65 wt%, 1 h, 45 °C)	Sulfated	[21–24]
Wood pulp	CNC: USDA Forest Products Laboratory (purchased from the University of Maine, USA)	*commercial CNC*	Sulfated	[25,26]
Tunicate cellulose pulp<br>*Extracted from Tunicates (Quimiac beach, France)*	-	(i) NaOH pretreatment (1 %, 10 h, boiling) – repeated twice<br>(ii) $NaClO_2$ pretreatment (0.3 %, pH 4.9, 2 h, 70 °C)<br>(iii) H_2SO_4 hydrolysis (65 wt%, 90 min, 40 °C)	Sulfated	[27]
Bleached softwood Kraft pulp	CNC: CelluForce Inc. (also procured from the University of Maine), Canada	*commercial CNC*	Sulfated	[28]
Cotton Linters	Cellulose: Buckeye Cellulose Corporation (Memphis, TN, USA)	H_2SO_4 hydrolysis (65 wt%, 30 min, 65 °C)	Sulfated	[29]

References

[1] J. Miller, *Nanocellulose: Producers, Products, and Applications: A Guide for End Users*, TAPPI Press, **2017**.
[2] Research and Markets ltd, *The Global Market for Nanocellulose - Research and Markets*, **2019**.
[3] Research and Markets ltd, *The Nanocellulose Report 2020 - Research and Markets*, **2020**.
[4] E. D. Cranston, *TAPPI International Conference on Nanotechnology for Renewable Materials conference presentation*, **2019**.
[5] J. Lockhart, in *Int. Conf. Nanotechnol. Renew. Mater.*, Chiba, **2019**.
[6] M. P. Andrews, T. Morse, *Method for Producing Functionalized Nanocrystalline Cellulose and Functionalized Nanocrystalline Cellulose Thereby Produced*, **2017**, US20170260298A1.
[7] S. McAlpine, J. Nakoneshny, *Production of Crystalline Cellulose*, **2017**.
[8] T. Retsina, V. Pylkkanen, A. van Heiningen, *Method for Vapor Phase Pulping with Alcohol, Sulfur Dioxide and Ammonia*, **2011**, US8038842B2.
[9] T. Retsina, V. Pylkkanen, A. R. P. van Heiningen, *Method for Vapor Phase Pulping with Alcohol and Sulfur Dioxide*, **2012**, US8268125B2.
[10] T. Retsina, V. Pylkkanen, *Method for the Production of Fermentable Sugars and Cellulose from Lignocellulosic Material*, **2011**, US8030039B1.
[11] T. Retsina, V. Pylkkanen, *Separation of Lignin from Hydrolyzate*, **2013**, US8585863B2.
[12] K. Nelson, T. Retsina, *TAPPI J* **2014**, *13*, 19–23.
[13] J.-L. Huang, C.-J. Li, D. G. Gray, *ACS Sustain. Chem. Eng.* **2013**, *1*, 1160–1164.
[14] A. R. Lokanathan, A. Nykänen, J. Seitsonen, L.-S. Johansson, J. Campbell, O. J. Rojas, O. Ikkala, J. Laine, *Biomacromolecules* **2013**, *14*, 2807–2813.
[15] L. R. Arcot, M. Lundahl, O. J. Rojas, J. Laine, *Cellulose* **2014**, *21*, 4209–4218.
[16] K. Heise, T. Koso, L. Pitkänen, A. Potthast, A. W. T. King, M. A. Kostiainen, E. Kontturi, *ACS Macro Lett.* **2019**, *8*, 1642–1647.
[17] H. Sadeghifar, I. Filpponen, S. P. Clarke, D. F. Brougham, D. S. Argyropoulos, *J. Mater. Sci.* **2011**, *46*, 7344–7355.
[18] M. A. Karaaslan, G. Gao, J. F. Kadla, *Cellulose* **2013**, *20*, 2655–2665.
[19] W. Du, J. Guo, H. Li, Y. Gao, *ACS Sustain. Chem. Eng.* **2017**, *5*, 7514–7523.
[20] L. Li, H. Tao, B. Wu, G. Zhu, K. Li, N. Lin, *ACS Sustain. Chem. Eng.* **2018**, *6*, 14888–14900.
[21] H. Tao, A. Dufresne, N. Lin, *Macromolecules* **2019**, *52*, 5894–5906.
[22] E. Sipahi-Sağlam, M. Gelbrich, E. Gruber, *Cellulose* **2003**, *10*, 237–250.
[23] S. Imlimthan, S. Otaru, O. Keinänen, A. Correia, K. Lintinen, H. A. Santos, A. J. Airaksinen, M. A. Kostiainen, M. Sarparanta, *Biomacromolecules* **2019**, *20*, 674–683.
[24] S. Imlimthan, A. Correia, P. Figueiredo, K. Lintinen, V. Balasubramanian, A. J. Airaksinen, M. A. Kostiainen, H. A. Santos, M. Sarparanta, *J. Biomed. Mater. Res. A* **2020**, *108*, 770–783.
[25] J. O. Zoppe, A. V. M. Dupire, T. G. G. Lachat, P. Lemal, L. Rodriguez-Lorenzo, A. Petri-Fink, C. Weder, H.-A. Klok, *ACS Macro Lett.* **2017**, *6*, 892–897.
[26] B. Risteen, G. Delepierre, M. Srinivasarao, C. Weder, P. Russo, E. Reichmanis, J. Zoppe, *Small* **2018**, *14*, 1802060.
[27] A. Villares, C. Moreau, B. Cathala, *ACS Omega* **2018**, *3*, 16203–16211.
[28] C. Tang, S. Spinney, Z. Shi, J. Tang, B. Peng, J. Luo, K. C. Tam, *Langmuir* **2018**, *34*, 12897–12905.
[29] F. Lin, F. Cousin, J.-L. Putaux, B. Jean, *ACS Macro Lett.* **2019**, *8*, 345–351.
